# Production and characterization of a broad-spectrum antimicrobial 5-butyl-2-pyridine carboxylic acid from *Aspergillus fumigatus* nHF-01

**DOI:** 10.1038/s41598-022-09925-z

**Published:** 2022-04-09

**Authors:** Vivekananda Mandal, Narendra Nath Ghosh, Prashanta Kumar Mitra, Sukhendu Mandal, Vivekananda Mandal

**Affiliations:** 1 Plant and Microbial Physiology and Biochemistry Laboratory, Department of Botany, University of Gour Banga, Malda, West Bengal India; 2 Department of Chemistry, University of Gour Banga, Malda, West Bengal India; 3 Department of Botany, Kalyani University, Nadia, West Bengal India; 4 Department of Microbiology, University of Calcutta, Kolkata, WB 700 019 India

**Keywords:** Biochemistry, Drug discovery, Microbiology

## Abstract

The present study aims at the production optimization, purification, and characterization of a potent broad-spectrum antimicrobial compound (AMC) produced by *Aspergillus fumigatus* nHF-01 (GenBank Ac. No. MN190286). The culture conditions were optimized for a higher amount of AMC. The AMC was solvent extracted and characterized by UV–Vis, FT–IR, ESI–MS, and ^1^H-NMR spectroscopy. The MIC, MBC and mode of action were determined against a set of Gram-positive and Gram-negative human pathogenic bacteria. Its antibiofilm, synergistic and cytotoxic effects were also tested. The putative target site of action was evaluated through in silico molecular docking study. The stain *A. fumigatus* nHF-01 produced the maximum AMC (5-butyl-2-pyridine carboxylic acid) in 2% MEB (w/v) and 4% YE (w/v) at pH 6.0 and 20 °C temperature with 100 rpm agitation for ten days. It caused complete lethality of the Gram-positive and Gram-negative human pathogenic bacteria at a 129 µg/mL dose by rupture and entire dissolution of cell integrity. It showed moderate antibiofilm activity and had a synergistic activity with streptomycin and additive effects with ciprofloxacin and vancomycin. It targets a respiratory enzyme, Quinol-Fumarate Reductase (1l0v), with the highest binding affinities. It had cytotoxicity against human lung carcinoma A549 cell line and was stable up to 100 °C. Thus, the study revealed that the strain *A. fumigatus* nHF-01 produces a potent broad-spectrum AMC 5-butyl-2-pyridine carboxylic acid that could be used against human food and topical pathogenic bacteria. This is the first report of such a compound produced from the *A. fumigatus*.

## Introduction

Natural products from plants and microbial sources, either as pure compounds or as standardized extracts, provide unlimited opportunities for new drugs discoveries due to unmatched chemical diversity^1^. Among the microbes, fungi have been a reservoir of isolation of many therapeutic drugs useful for ameliorating and curing many physiological, metabolic, and genetic diseases such as Alzheimer's disease, cancer, diabetics, hypercholesterolemia, etc.^2–4^. Since discovering the life-supporting drug, Penicillin, the first *β*-lactam antibiotic, by Sir Alexander Fleming in the 1920s, lots of progress have been developed in the antibacterial compounds production, process development and characterization of active molecules. Due to the ever upsurge of antimicrobial resistance among the pathogenic microbes, nowadays, it is challenging to find new antimicrobial compounds against resistant pathogenic strains^5^.

Among the members of fungi, compared to the other genera, *Aspergillus* has been as widely explored for its applications in basic genetic studies and various industries, such as foods, detergents, textiles, cosmetics, and pharmaceuticals for the production of numerous valuable extracellular enzymes, organic acids, and secondary metabolites of biotechnological importance^6^. Genome projects have been completed for *A. fumigatus*, *A. nidulans*, *A. niger* and *A. oryzae* and it revealed that the species contains a genome of 28–30 Mb^7,8^. The whole-genome sequencing has revealed that fungi could produce a far greater number of secondary metabolites that have been isolated. The genome mining of secondary metabolites produced by *A. fumigatus*, *A. nidulans*, *A. niger*, and *A. terreus* revealed unique biosynthetic gene clusters and biosynthetic pathways^9^. Several studies reported that *Aspergillus* species significantly produce many life-supporting drugs comprising mainly antibacterial, antifungal, antiviral, cytotoxic, antioxidants, against AIDS, surgery or transplantation surgery, and many more such diseases^10–13^. Therefore, it became apparent that each of these fungi possesses considerable potential as expression hosts for producing numerous small molecules and heterologous proteins^14^.

*Aspergillus* spp. are cosmopolitan micro-fungi growing in different ecological niches, including extreme environments. They are known to grow in decaying substances, endophytes, and even extreme saline or cold environment^15–17^. For example, the endophytic strain of *A. fumigatus,* isolated from *Moringa oleifera,* produces antimicrobial and antibiofilm compounds and exhibited excellent antiproliferative activity against different cancer cell lines with the IC50 values of 0.061–0.072 mg/mL^16^ while *A. fumigatus* EFBL, isolated from *Catharanthus roseus*, produces epothilone (21.5 μg/g biomass), which has potent antiproliferative activity against tumour cells HepG-2, MCF-7 and LS174 T at IC_50_ values 6.4, 8.7 and 10.21 μM, respectively, by stabilizing their microtubule arrays and arresting their cellular division at the G2-M phase^18,19^. A mutant strain of *A. fumigatus* could produce anticancer drug paclitaxel in immobilized calcium alginate gel beads. Another endophytic *A. fumigatus* CY018, from the leaf of *Cynodon dactylon,* produces two new metabolites, named asperfumoid and asperfumin, inhibitory to *Candida albicans* with MICs of 75.0 µg/mL^20^. Moreover, certain *Aspergillus* spp. are reported from deep-sea sediments or symbionts to marine animals, producing various bioactive compounds^21–24^. For example, a deep-sea derived fungal *A. fumigatus* SCSIO produces two new alkaloids, fumigatosides E and F, showing significant antifungal activity with MIC at 1.56 µg/mL, and antibacterial activity against *Acinetobacter baumanii* ATCC 19606 with a MIC value of 6.25 µg/mL, respectively^17^.

In addition to the small molecule AMCs, the species of *Aspergillus* are known to produce valuable peptides of therapeutic use. For example, an extracellular thermostable peptide (MFAP9, ∼ 3 kDa) purified from marine *A. fumigatus* BTMF9 exhibited strong antibacterial and antibiofilm activity against *Bacillus circulans* (NCIM 2107) and *B. pumilus* with MIC and MBC values of 0.525 μg/mL and 4.2 μg/mL, respectively^25^, while, the proteins from *A. fumigatus* DSM819 have been used in the green synthesis of silver nanoparticles (AgNPs) for its application as an antimicrobial finishing agent in textile fabrics against pathogenic microorganisms (*B. mycoides, C. albicans* and *E. coli*)^26^.

Therefore, from the above literature studies, it appears that the strains of *A. fumigatus* play significant roles as model organisms in basic research and "cell factories" for producing various industrial products of human use. In this connection, the present study hypothesizes to characterize the potent broad-spectrum AMC produced by *A. fumigatus* nHF-01 in a submerged fermentation system and evaluate its detailed antibacterial efficacy against a large number of bacterial pathogens associated with food and topical pathogenesis.

## Results

### The strain *Aspergillus fumigatus* nHF-01: a azole sensitive strain

The strain *A. fumigatus* nHF-01 could grow in different media like Malt extract broth (MEB), Czapek dox broth (CDB), and Cornmeal broth (CMB) at pH ranges from 3.0 to 10.0 and temperature ranges from 20 to 45 °C^13^. To check the health risk, an antimicrobial drug resistance study was done. The MIC of eight different antifungal drugs is shown in Table 1. The study shows that among these drugs, Luliconazole exhibited the best in vitro activity with MIC values of 0.25 µg/mL, while Fluconazole was the less active with MIC values of 10 mg/mL (Supplementary Table S1).

**Table 1:** MIC of antifungal drugs for *Aspergillus fumigatus* nHF-01 using agar well diffusion method.

Sl. no.	Antifungal drugs	MIC values
1	Amphotericin B	< 20 µg/mL
2	Clotrimazole	500 µg/mL
3	Fluconazole	10 mg/mL
4	Gresiofulvin	> 1 mg/mL
5	Itraconazole	31.25 µg/mL
6	Ketoconazole	700 µg/mL
7	Luliconazole	0.25 µg/mL
8	Terbinafine	8 mg/mL

### Optimum culture conditions for AMC production

**Figure 1:**
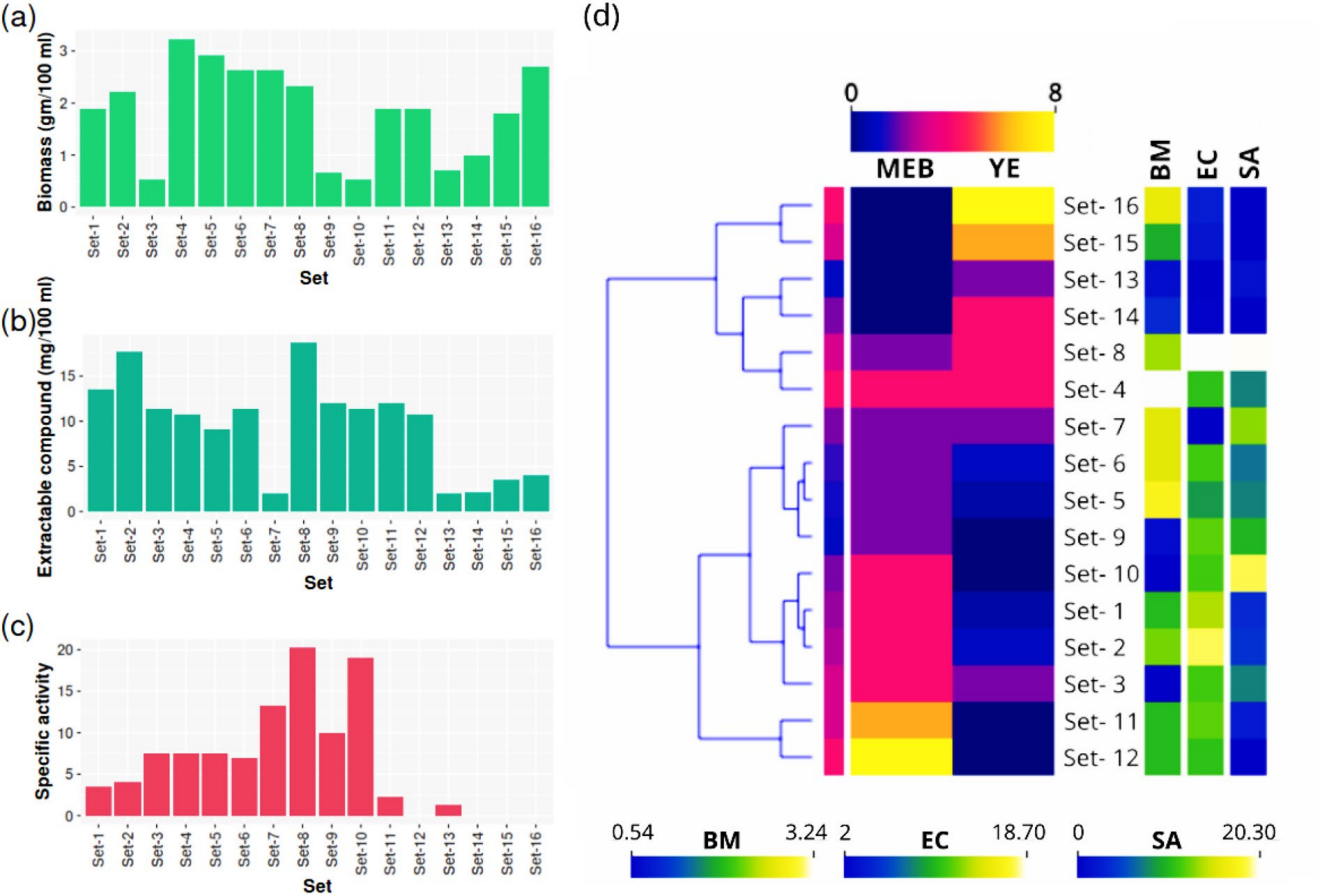
Bar plots (**a**–**c**) of production parameters from different sets of media composition. (**a**) biomass (BM), (**b**) extractable compound (EC), (**c**) specific activity (SA). (**d**) Heatmap showing clustering the similar media based on production results.

**Figure 2:**
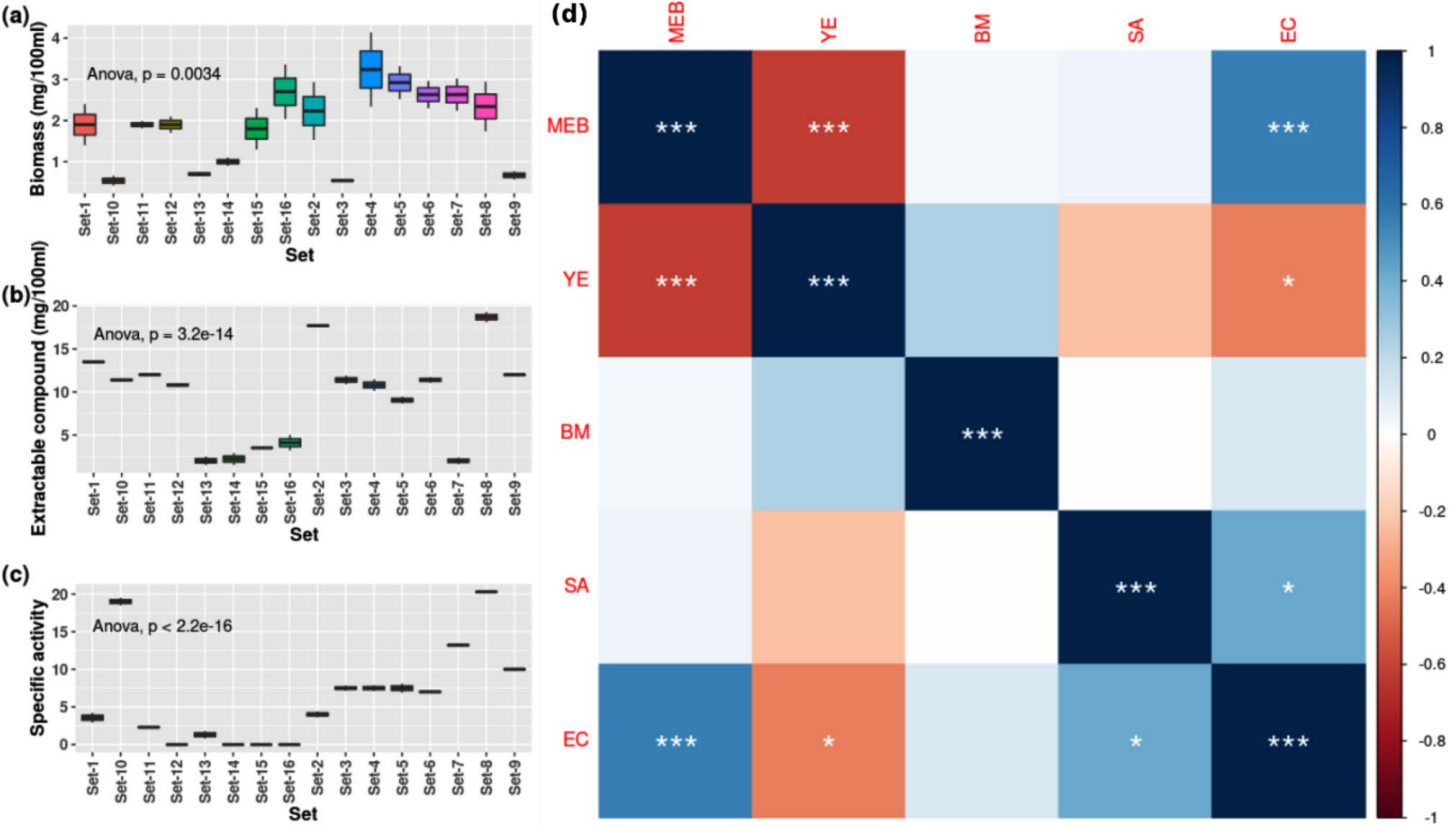
Box plots (**a**–**c**) represent the data distribution range of biomass (BM), extractable compound (EC), specific gravity (SA) and variation between experimental sets. Correlation matrix (**d**) represents the correlation between parameters [Colour scale bar at right representing correlation from negative (− 1) to positive (+ 1). Significance stars representing significance level of correlation (*p < 0.05, **p < 0.01, ***p < 0.001)].

Among the different culture media, the highest amount of AMC was produced in MEB and extractable in DCM solvent, producing the highest growth inhibition zone of 25 to 30 mm, at a 15 mg/mL concentration, comparable to streptomycin (20 µg/mL) against the tested bacteria (Supplementary Table S2; Supplementary Fig. S1). It produced suitable biomass of mycelia (an average 1.334 g/100 mL culture) and AMC (an average 10.5 mg/100 mL culture). In comparison, the other media produced a negligible antibacterial activity though they had a high biomass production. The suitable pH and temperature range were at pH 6.0, 20 °C for 10 days incubation in 100 rpm shaking (Supplementary Fig. S2). A combinational study with MEB and yeast extract (YE) showed that a mixture of 2% MEB and 4% YE (w/v) produced the highest specific activity (Table 2; Fig. 1a–c). Set-4 produced the highest amount of biomass among the variants, while Set-8 produced the highest extractable compound and specific activity. The Set-7 produced a good amount of biomass but a very low extractable compound with high specific activity. Moreover, Set-12, Set-14, Set-15, Set-16 were found to produce a low-moderate extractable compound with no specific activity. From the heat map (Fig. 1d), it was observed that a high concentration of YE produces poor results in terms of the extractable compound and specific activity; according to the interest of this study, Set-4 and Set-8 was clustered together for producing nearly similar results. A significant difference was found between sets (1 to 16) for biomass (p = 0.0034, df = 15), extractable compound (p = 3.2e−14, df = 15) and specific activity (p < 2.2e−16, df = 15) (Fig. 2a–c), indicating a prominent effect on fungal culture due to mixture of MEB and YE. A significant positive correlation was found between MEB:EC (p < 0.001) and SA:EC (p < 0.05), and significant negative correlation was found between YE:EC (p < 0.05) (Fig. 2d).

**Table 2:** Table presenting experimental details.

Set	Predictor variables		Response variables		
	MEB%	YE%	Biomass	Extractable compound	Specific activity
Set-1	4	0.5	1.9 ± 0.5	13.5 ± 0.05	3.57 ± 0.66
Set-2	4	1	2.23 ± 0.7	17.7 ± 0.1	4.01 ± 0.4
Set-3	4	2	0.54 ± 0.03	11.4 ± 0.5	7.5 ± 0.33
Set-4	4	4	3.238 ± 0.9	10.8 ± 0.66	7.5 ± 0.39
Set-5	2	0.5	2.92 ± 0.4	9.05 ± 0.4	7.5 ± 0.6
Set-6	2	1	2.63 ± 0.33	11.4 ± 0.33	7 ± 0.1
Set-7	2	2	2.63 ± 0.39	2 ± 0.39	13.21 ± 0.12
Set-8	2	4	2.34 ± 0.6	18.7 ± 0.6	20.3 ± 0.05
Set-9	2	0	0.67 ± 0.1	12 ± 0.1	10 ± 0.1
Set-10	4	0	0.54 ± 0.12	11.4 ± 0.12	19 ± 0.5
Set-11	6	0	1.9 ± 0.09	12 ± 0.09	2.3 ± 0.09
Set-12	8	0	1.9 ± 0.2	10.8 ± 0.2	0
Set-13	0	2	0.7 ± 0.05	2 ± 0.5	1.3 ± 0.5
Set-14	0	4	1 ± 0.1	2.2 ± 0.7	0
Set-15	0	6	1.8 ± 0.5	3.5 ± 0.03	0
Set-16	0	8	2.7 ± 0.66	4.1 ± 0.9	0

Here, the values of the response variables are the average of duplicate trials ± SE.

**Figure 3:**
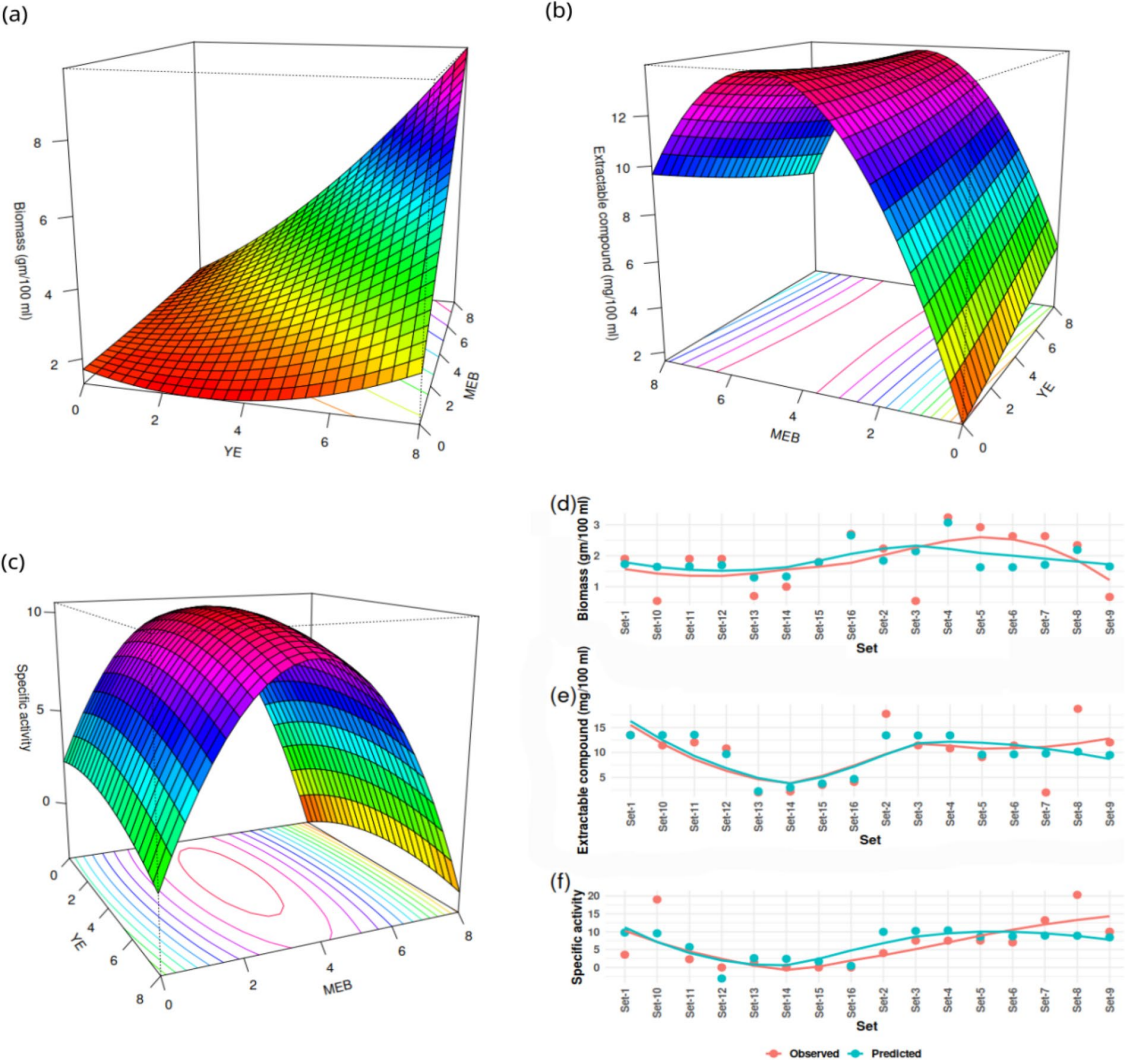
Surface plots (**a**–**c**) show effects of interaction between MEB and YE on biomass (**a**), extractable compound (**b**), specific activity (**c**). Comparison between predicted values from RSM models for biomass (**d**), extractable compound (**e**) and specific activity (**f**) with observed values are presented in dot plots, and lines are comparing the mean values.

From surface plots (Fig. 3a–c), the interaction between MEB and YE was determined. Higher concentrations of MEB and YE at equal proportions induces higher biomass production (Fig. 3a). The highest production of the extractable compound was produced between MEB concentrations 4 to 6; YE was found to contribute less in the production of the extractable compound and was controlled mainly by the concentration of MEB (Fig. 3b). MEB concentration 4% and YE concentrations between 2 to 6% were most suitable for achieving the highest specific activity (Fig. 3c). From the dot plots (Fig. 3d–f) comparing predicted values with observed values, it can be observed that the models predicting response are fitted quite well with the observed values and can be used for further development. Although the models fitted well (Fig. 3d–f), none of the models (for biomass, extractable compound, specific activity) found significant (at 0.05 alpha level) (Table 3). The R-squared and adjusted R-squared values turned out to be moderate to low; probably, a better experimental design can improve the design and R-squared values. Model details and coefficients are given below in Table 3.

**Table 3:** Table presenting summarized details of RSM models.

Predictor variables	MEB, YE											
Response variables	Biomass, extractable compound, specific activity											
Coefficients of variables and interactions												
	Biomass				Extractable compound				Specific activity			
	Estimate	Std. Error	t value	p-value	Estimate	Std. Error	t value	p-value	Estimate	Std. Error	t value	p-value
Intercept	1.6874	1.537	1.0978	0.298	1.60465	6.8692	0.2336	0.82	2.2997	9.7593	0.2356	0.8185
MEB	− 0.0235	0.6922	− 0.034	0.9735	4.9185	3.0934	1.59	0.1429	4.3022	4.3948	0.9789	0.3507
YE	− 0.3003	0.7169	− 0.4188	0.6842	0.2981	3.2039	0.093	0.9277	0.2858	4.5519	0.0628	0.9512
MEB × YE	0.1115	0.143	0.7801	0.4534	− 0.0884	0.639	− 0.1383	0.8928	0.0445	0.9078	0.049	0.9619
MEB^2^	0.003	0.0712	0.0426	0.9668	− 0.4889	0.3183	− 1.5363	0.1555	− 0.6221	0.4522	− 1.3757	0.1989
YE^2^	0.0527	0.0751	0.0751	0.4989	0.011	0.3354	0.0329	0.9744	− 0.0646	0.4766	− 0.1355	0.8949
R-squared	0.2602				0.5739				0.424			
Adjusted R-squared	− 0.1098				0.3609				0.136			
DF	10				10				10			
p-value	0.6339				0.08557				0.2813			

Polynomial equations were derived from the respective RSM models (biomass, extractable compound and specific activity) to generate the surface plot. The mathematical formulas used are as follows:

$${\mathbf{Biomass:}} {\text{Y}} = { 1}.{6874} - 0.0{235} \times {\text{X 1 }} - 0.{3}00{3} \times {\text{X 2 }} + 0.00{3} \times {\text{X 1 2 }} + 0.0{527} \times {\text{X 2 2 }} + 0.{1115} \times {\text{X 1 }} \times {\text{X 2}}$$

$${\mathbf{EC:}} {\text{Y}} = { 1}.{6}0{465} + {4}.{9185} \times {\text{X 1 }} + 0.{2981} \times {\text{X 2 }} - 0.{4889} \times {\text{X 1 2 }} + 0.0{11} \times {\text{X 2 2 }} - 0.0{884} \times {\text{X 1 }} \times {\text{X 2}}$$

$${\mathbf{SA:}} {\text{Y}} = { 2}.{2997} + {4}.{3}0{22} \times {\text{X 1 }} + 0.{2858} \times {\text{X 2 }} - 0.{6221} \times {\text{X 1 2 }} - 0.0{646} \times {\text{X 2 2 }} + 0.0{445} \times {\text{X 1 }} \times {\text{X,}}$$

where, X_1_ = MEB, X_2_ = YE, X_1_^2^, = MEB^2^, X_2_^2^ = YE^2^, X_1_ × X_2_ = MEB × YE.

### Chromatographic purification of the AMC

The separation of constituents of the DCM extract by TLC revealed three distinct spots with *Rf* values of 0.33 (C2), 0.53 (C3) and 0.63 (C4), as shown in Supplementary Fig. S3a. The bioassay of the TLC plate and sub-fraction from TLC scraped showed that the fraction with *Rf* value 0.63 (C4), out of the three spots, was the potentially active AMC (Supplementary Fig. S3b–e). HPLC chromatogram of the active DCM fraction (C4) showed the presence of three peaks with retention times 3.725, 4.772 and 5.083 with peak area 17,986,632, 16,175,955 and 43,911,062, respectively (Supplementary Fig. S3f). The peak height 503,341, with a retention time of 3.725, had higher efficacy than the other 4.772 and 5.083 retention times.

### Characterization of active compound

#### UV–Vis and FT–IR spectrophotometric analysis

The UV–visible spectrum of pure DCM fraction showed three major peaks (Supplementary Fig. S4a) with λ_max_ values at 229 nm, 270 nm, and 358 nm. The FT-IR analysis showed peak values at 804.3 cm^−1^, 929.6 cm^−1^ and 1026 cm^−1^ (Supplementary Fig. S4b), which indicate the functional group's alkenes (C=C), carboxylic acids (O–H), and amines (C–N), respectively^27^. The values 2860.4 cm^−1^, 2931.8 cm^−1^ and 2966.5 cm^−1^ indicate alkanes (C–H). The peak values between 2300 and 2400 cm^−1^ indicate the CO_2_ rich in the spectrum. Some more recognizable intense peak values in the spectrum show some unusual band pattern among the FT–IR spectra of organic molecules. Therefore, more in-depth molecular characterization would help decipher the molecule's novelty.

#### GC–MS, ESI–MS and NMR analysis of pure compound

According to mole %, the GC-chromatogram showed a spectral match with 5-butyl 2-pyridine carboxylic acid, (mole% 5.15, RT-18.996 min) (Supplementary Table S3). The high-resolution ESI–MS spectrum (Supplementary Fig. S4c) showed a molecular ion peak at *m/z* 180.1047, corresponding to the compound 5-butyl-2-pyridine carboxylic acid with an empirical formula of C_10_H_13_NO_2_. The ^1^H NMR spectrum (Supplementary Fig. S4d) of the purified compound exhibited signals at 1.30, 1.68, 2.77, and as a multiplet and two triplets, respectively, for a –CH_2_–CH_2_–CH_2_– grouping, plus three aromatic protons of benzene ring at δ 8.20, 8.18 and 7.84 and signals at δ 0.8916 (triplet) for the presence of methyl (CH_3_) which is concordant with the previous observations^28^. Thus, the ESI–MS and ^1^H NMR spectral data confirmed the potential AMC as 5-butyl-2-pyridine carboxylic acid with a molecular formula of C_10_H_13_NO_2_ (Supplementary Fig. S4e).

### Phytochemical screening, solubility and thermo-stability of the compound

Phytochemical screening of the pure fraction showed the presence of alkaloids (Supplementary Table S4). Alkaloids are heterogenous natural nitrogen-containing organic compounds used to treat bacteria and serve as scaffolds for essential antibacterial drugs^29^. The thermal treatment on the compound showed stability up to 100 °C; however, it lost its activity at autoclave temperature. The compound had a solid appearance with a deep brownish colour and was soluble in various polar and non-polar solvents, viz*.* *n*-hexane, diethyl ether, DCM, ethyl acetate, methanol, DMSO and chloroform.

### The MIC and MBC values and effect on bacterial growth, viability and cellular integrity

**Figure 4:**
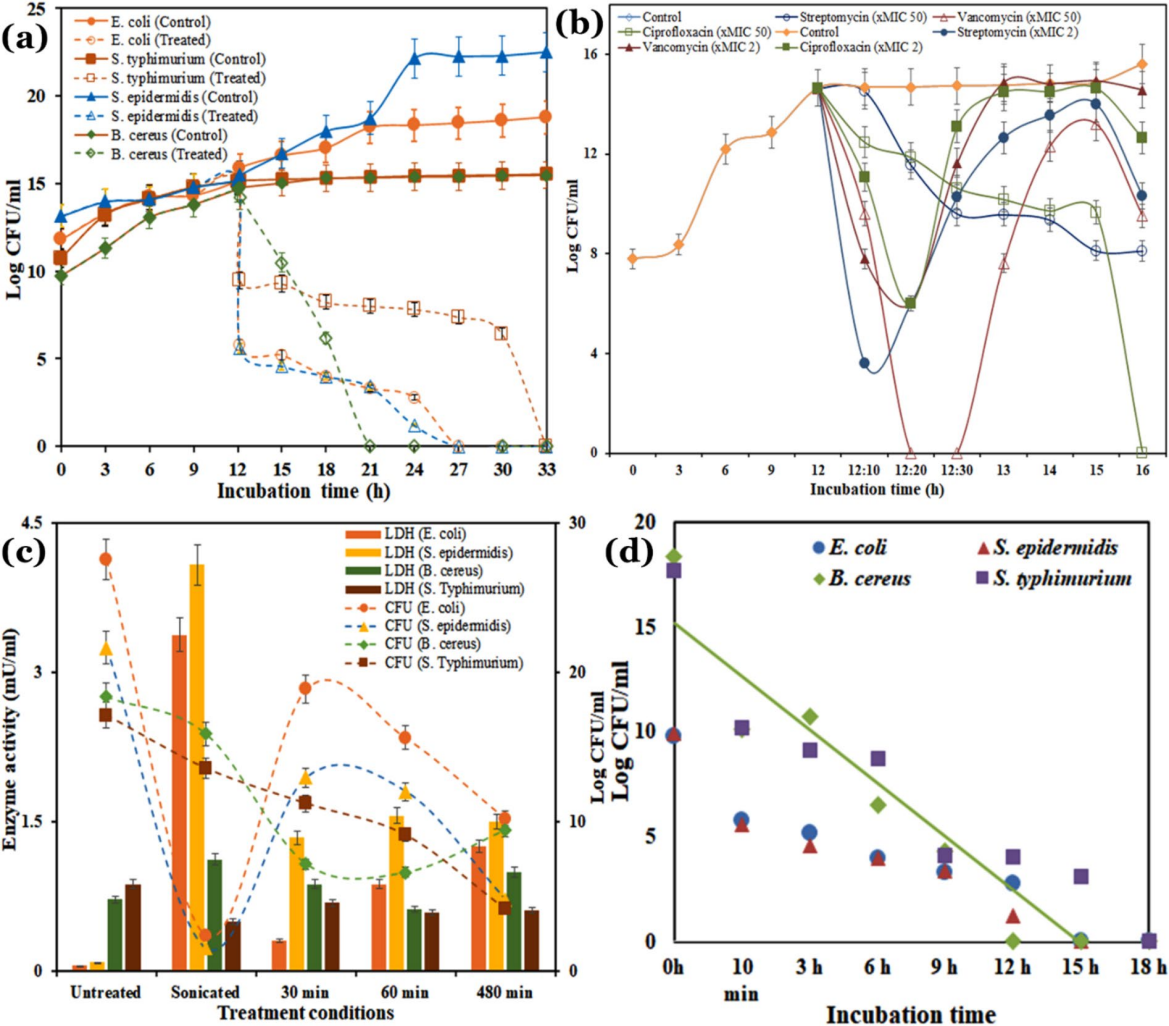
Mode of action of the 5-butyl-2-pyridine carboxylic acid on viability, cellular integrity and time-kill kinetics. (**a**) Effect on bacterial viability; (**b**) Effect of different antibiotics on *E. coli*; (**c**) Effect on cellular integrity (LDH) and bacterial viability (CFU), and (**d**) time-kill endpoint.

The purified 5-butyl-2-pyridine carboxylic acid produced a potent antibacterial activity against the tested microorganisms. The MIC and MBC values were variable among the strains. The MIC values ranged from 0.069 ± 0.0034 to 1.12 ± 0.052 mg/mL and from 8.925 ± 0.39 to 17.85 ± 0.78 mg/mL; while the MBC ranged from 8.925 ± 0.40 to 17.85 ± 0.776 mg/mL and from 0.069 ± 0.0034 to 0.139 ± 0.0065 mg/mL against human pathogenic Gram-positive and Gram-negative bacteria, respectively (Table 4). The effect of the active compound on growth and viability against human pathogenic Gram-positive bacteria (*B. cereus* and *S. epidermidis*) and Gram-negative bacteria (*E. coli* and *S. enterica* serovar Typhimurium) showed that at concentration 0.139 and 17.85 mg/mL, respectively it decreased the viability sharply within 15 min of the incubation period (Fig. 4a). Thus, a rapid decrease in the growth and viability of treated bacterial cells indicated that the active compounds had a strong bactericidal mode of action against the tested pathogens^30,31^. A comparative growth inhibition study with standard antibiotics, ciprofloxacin, streptomycin and vancomycin at MICx2 and MICx50 doses showed that all three antibiotics caused a rapid loss of viability within 30 min of treatment (Fig. 4b, and Supplementary Fig. S6). However, surprisingly the CFU/mL of the tested bacteria regained viability after 60 min. This observation indicates that the organisms either developed transient resistance to the antibiotics by changing the target site of action or enhancing the cellular transport or the efflux mechanism of the antibiotics. On the contrary, these results emphasize the potency of the new active compound from the *A. fumigatus* nHF-01 could be a potential lead drug in future antimicrobial therapy.

**Table 4:** MIC and MBC values of the 5-butyl-2-pyridine carboxylic acid against pathogenic bacteria.

Gram status	Bacteria	Concentration (mg/mL)	
		MIC	MBC
Gram-positive	*B. cereus* MTCC 1272	0.069 ± 0.0034	0.139 ± 0.006
	*B. subtilis* MTCC 411	0.279 ± 0.012	0.557 ± 0.021
	*E. faecalis* MCC 2041T	8.925 ± 0.41	17.85 ± 0.85
	*M. smegmatis* mc^2^ 155	4.46 ± 0.20	8.925 ± 0.379
	*S. aureus* MTCC 96	0.557 ± 0.022	1.12 ± 0.052
	*S. epidermidis* MTCC 3086	8.925 ± 0.39	17.85 ± 0.78
	*S. mutans* MTCC 890	0.557 ± 0.025	1.12 ± 0.038
Gram-negative	*E. coli* MTCC 723	8.925 ± 0.40	17.85 ± 0.776
	*E. coli* ATCC DH5α	4.46 ± 0.175	8.925 ± 0.354
	*K. pneumonia* MTCC 7407	0.557 ± 0.021	1.12 ± 0.019
	*P. aeruginosa* MTCC 741	0.557 ± 0.025	1.12 ± 0.023
	*S. enterica* serovar Typhimurium MTCC 98	0.069 ± 0.0034	0.139 ± 0.0065
	*V. parahaemolyticus* MTCC 451	1.12 ± 0.019	2.23 ± 0.10

Here, the values are the average of triplicate trials ± SE, while the 'MIC' and 'MBC' indicate the values of the active compound that caused significant loss of viability of the organisms.

**Figure 5:**
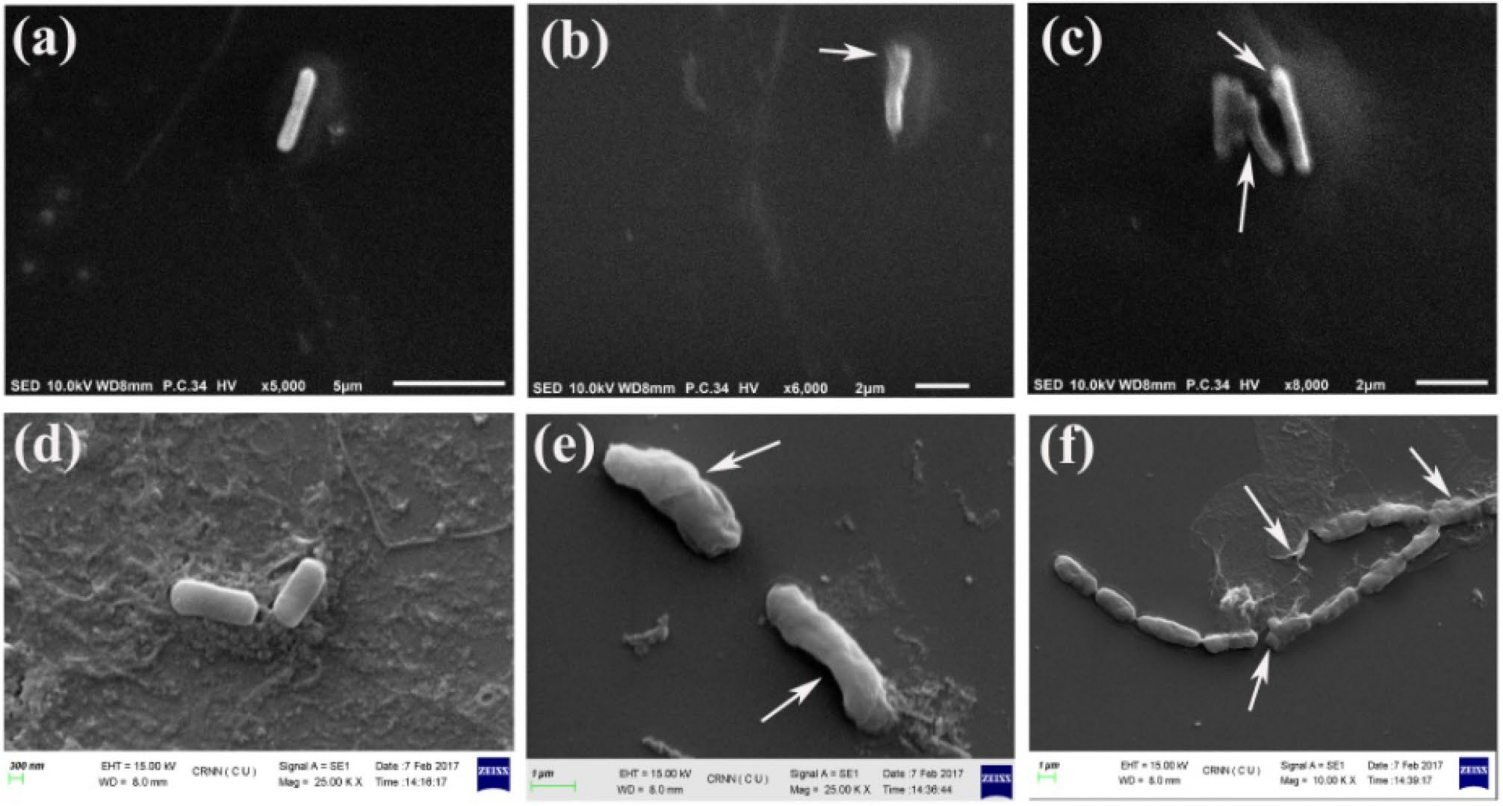
SEM photomicrographs of *B. cereus* and *E. coli* cells treated with a 5-butyl-2-pyridine carboxylic acid. (**a**,**d**) Untreated control cells of *B. cereus* and *E. coli* (magnification: × 5000, × 2500 K, respectively); (**b**,**c**) treated *B. cereus* cells after 30 min and after 3 h incubation (magnification: × 6000, × 8000, respectively). (**e**,**f**) treated *E. coli* cells after 30 min and 3 h incubation (magnification: × 2500 K, × 1000 K, respectively). The significant morphological changes have been marked with white arrows.

Lactate dehydrogenase (LDH) is an essential cytoplasmic enzyme of all living cells. In the presence of the pure active compound, the cellular integrity had lost with the gradual increase in LDH activity (Fig. 4c). A subsequent gradual decrease in the colony-forming unit was observed in all the strains tested. The positive control (sonication) showed that it had high LDH with the lowest CFU values. The time-kill curve showed that after 12 h of treatment, the compound at a MIC × 2 dose caused zero viability from the initial Log CFU values (Fig. 4d). This indicates that the compound had a bactericidal activity with absolute lethality in the treated cells. The effect of the active compound on *B. cereus* and *E. coli* showed remarkable changes in the morphology (Fig. 5). It was found that in comparison to the untreated cells (Figs. 5a and 4d), the treatment after 30 min caused minute cell wall to rupture (Fig. 5b,e), and at 3 h of treatment, it caused drastic changes in cell morphology like the formation of blebbing, notch, rupture of the entire cell walls, and entire dissolution of cell integrity (Fig. 5c,f). This indicates that the compound is causing lysis of the bacterial cell wall resulting in a rapid decrease in cell viability.

### Antibiofilm and biofilm destabilization assay

**Figure 6:**
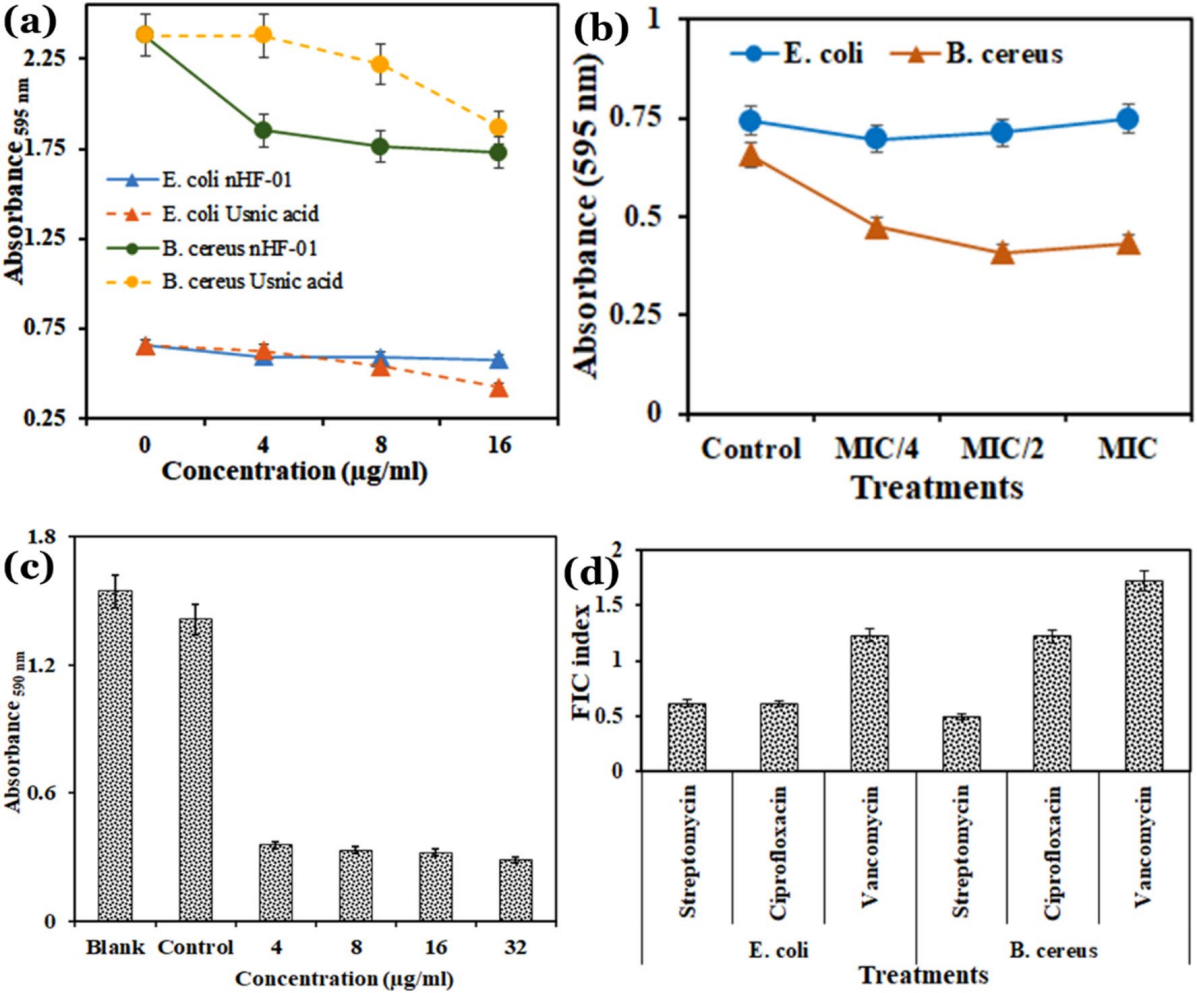
Effect of the 5-butyl-2-Pyridinecarboxylic acid on (**a**) biofilm inhibition; (**b**) biofilm destabilization; (**c**) cytotoxicity assay against A549 cell line; (**d**) synergistic effects with three common antibiotics, ciprofloxacin, streptomycin and vancomycin.

Biofilm is a complex association of microorganisms formed on solid and liquid systems. *B. cereus* and *E. coli* are found to be significant organisms residing on food commodities, on surfaces of package materials, and even inside the human body, forming a toughened matrix. Many potential AMCs are found ineffective in this state of the microbes. The present study revealed that the active compound showed moderate inhibitory properties on the biofilm-forming *E. coli* and *B. cereus* compared to the standard antibiofilm compound Usnic acid (Fig. 6a). The active compound at a concentration of 4 µg/mL and 129 µg/mL showed 22.30% of biofilm inhibition against *B. cereus* and *E. coli,* respectively, compared to control. The percentage of such inhibition is concentration-dependent, and it was 45.38% and 65.18% at a concentration of 517 µg/mL. The standard antibiofilm drug usnic acid showed a notable antibiofilm activity at 10 µg/mL and 64 µg/mL against *E. coli* and *B. cereus,* which is comparable to 20 µg/mL, and 4 µg/mL of the nHF-01 active compound. So the study revealed that the active compound 5-butyl-2-pyridine carboxylic acid has the potential to inhibit both the planktonic and biofilm stages of the Gram-positive and Gram-negative bacterial strains. The biofilm destabilization assay also showed that it could destabilize the preformed biofilm of the target pathogens (Fig. 6b).

### Cytotoxicity and synergistic effect with different antibiotics

The compound 5-butyl-2-Pyridinecarboxylic acid showed cytotoxicity against the tested human A549 cell line (Fig. 6c). The synergistic effects of 5-butyl-2-pyridine carboxylic acid with three conventional antibiotics (ciprofloxacin, streptomycin and vancomycin) showed that all combinations demonstrated synergistic, partiality synergistic and additive effects against the tested bacteria (Fig. 6d). It showed synergistic activity with streptomycin against *B. cereus*, whereas it has additive effects with ciprofloxacin and vancomycin. While it showed partial synergistic activity with streptomycin and ciprofloxacin in *E. coli*, an additive effect with vancomycin (Fig. 6d).

### Molecular modelling

Molecular docking between the active compound, 5-butyl-2-Pyridinecarboxylic acid, and a large number of target site action showed (Supplementary Table S5) that the three different respiratory target enzymes viz*.* Quinol-Fumarate Reductase (QFR, 1kf6), Quinol-Fumarate Reductase (1l0v) and quinone oxidoreductase (SQR) SdhB His207Thr mutant (2wp9) of *E. coli* had the highest binding affinities. Among these, it showed the highest binding affinities with Quinol-Fumarate Reductase (1l0v) (docking score − 7.1). The binding mode of the compound with these enzymes are illustrated in Supplementary Fig. S5a–i, and it formed 2 H-bonds with SER36, ALA12 residues of Quinol-Fumarate Reductase, 2 H bonds with ALA12, SER43 residues of Quinol-Fumarate Reductase with Menaquinol and 5 H-bonds with GLY 402, ARG286, ARG399, HIS 354 residues of succinate: quinone oxidoreductase, respectively, as shown in 3D and 2D contour plots in Supplementary Fig. S5, along with respective H-bonds.

## Discussion

### *A. fumigatus* nHF-01 and AMC production

The species of *Aspergillus* sp. have been reported to have pathogenicity to human beings^32^. To check such health risks, a drug resistance/sensitivity test of an isolate to life-supportive drugs is very much crucial. Among these, azole resistance is one of the leading concerns. The results show that the present strain *A. fumigatus* nHF-01 is sensitive to such azole and other antifungal drugs; thus, it is assumed that the handling of the strain for large scale AMC production is less risky and safe (Supplementary Table S1). The culture conditions and incubation period is crucial consideration for AMC production. The AMC production by the strain showed that the MEB and YE (low-cost fermenting substrates) triggered a high amount of AMC when grown at low temperatures (20 °C) with mild acidic (pH 6.0) fermentation conditions. It did not produce AMC at neutral to alkaline pH (8.0–12.0) and strong acidic pH (3.0–5.0) though it has ample mycelial biomass. The results indicate that a unique H^+^ balance might induce the associated molecules or genes to produce AMC. The influence of temperature on antibiotic production varies from strain to strain. Generally, fungi are grown at 28 °C at pH 5.6–6.5. Effect of incubation temperatures and pH showed that *A. fumigatus* nHF-01 produced the maximum stable and effective AMC with inhibition zone (30–32 mm) in pH 6.0 at 20 °C while at 28 °C, 37 °C and 45 °C significantly less inhibition zone (7–23 mm) was observed at the same concentration of extractable mass. Low temperature accelerated the metabolite production by the fungus while high temperature slowed down. The subsequent microscopic observation shows that at 20 °C, *A. fumigatus* nHF-01 had significantly more sporulation with restricted growth, while at > 20 °C, it had a puffy and velvety appearance less sporulation, no prominent vesicle was found in both liquid and solid media (Supplementary Fig. S1), as reported earlier^13^. This indicates that the cultural conditions of this organism are pretty unusual, and restricted mycelial growth with more sporulation is ideal for this organism for AMC production. A similar observation was reported in a marine fungus *A. ustus* MSF3 that produced AMC in 45% Sabouraud dextrose broth (SDB) with carbon (glucose)—nitrogen (yeast extract) ratio of 3:2 at 20 °C temperature at 7 days in solid culture^33,34^. Compaore et al*.*^35^ and Agastian et al*.*^36^ reported that *A. fumigatus* produces fumagillin and gliotoxin optimally in a synthetic media condition (supplemented with yeast extract, lactose and other carbon sources) for 6–8 days of incubation at 37 °C, pH 7.0, at 150 rpm, and extractable in acetonitrile and methanol. It was also observed that *A. fumigatus* nHF-01 produced maximum AMC at 10th day with an average diameter of 19–23 mm inhibition zone at 15 mg/mL concentration, while the 8th and 12th days period produced significantly less antimicrobial activity at the same concentration (Supplementary Fig. S2). Moreover, no inhibition zones were observed on the 5th, 15th and 20th days of incubation. This indicates that the organisms’ physiological status, like cell age and the media’s nutritional status, might trigger the organism to produce such AMC. In addition, a sudden drop in antimicrobial content on the 12th day indicate that the organism could produce some degradative enzymes to impede the activity of the antimicrobial compounds. On the other hand, the compounds in cell-free supernatant or after extraction with DCM were stable for more than 36 months. So, the activity loss is not due to compound stability but degradative molecules’ production. In addition to nutrient conditions, circulatory agitation also helps aeration in the culture medium. It produced a higher antimicrobial compound (0.688 mg/mL) at an optimum agitation speed of 100 rpm than non-agitation (0.38 mg/mL) condition. It is hypothesized that this fungal organism gets its optimum sheerness to spread or grow its mycelia and gain its optimum gas balance (O_2_/CO_2_) inside the culture system to produce the metabolite optimally. It was also observed that AMC produced by the mycelia was exclusively released in the culture broth and no further extracts were recoverable from the dried mycelia. This is unique from the industrial point of view. Considering all these findings, for AMC production by *A. fumigatus* nHF-01, certain unique fermentation conditions are of the utmost need for the strain and all these parameters would guide designing the RSM model for large scale production at an industrial scale. A similar culture condition-dependent metabolite profiling of *A. fumigatus* with antifungal activity study was done by Kang et al.^37^ and they revealed that *Aspergillus* sections Fumigati (*A. fumigatus*), Nigri (*A. niger*), and Flavi (*A. flavus, A. oryzae*, and *A. sojae*) have separate culture conditions requirement regardless of culture medium.

### AMC characterization and its structure–function relations on bacterial cells: Planktonic and biofilm stages

The spectroscopic studies revealed that the molecular structure of the AMC is 5-butyl-2-pyridine carboxylic acid (also known as Fusaric acid, FA or 5-butyl-2-picolinic acid). The molecule and its analogues exhibited moderate antimicrobial activities^38^, including growth inhibitors of *E. coli*^39^, and act as quorum sensing^40^. It is also reported from species of *Fusarium*^41^ and *Gibberella fugikuroi*^39^. Moreover, previous studies also show that it inhibits dopamine beta-hydroxylase enzymes that convert dopamine to norepinephrine and inhibits cell proliferation and DNA synthesis^40^ anti-tumour activity on heme enzymes^39^. Structure–activity co-relation indicates that 2-pyridine carboxylic acid and its derivatives act as a bidentate chelating agent that effectively chelates metals in metal-containing protein complexes and enzymes required for growth replication or inflammatory response and thereby used to treat cancer. So the novelty of the present study is that the strain *A. fumigatus* would provide an easy biological source for large scale production of 5-butyl-2-pyridine carboxylic acid. The AMCs with such broad-spectrum activities is very limiting to the list of antimicrobial drugs in pharmaceutical industries. The study shows that the MIC and MBC values were more active against a broad range of food and waterborne pathogens that cause fatal food poisoning, typhoid fever, tuberculosis, and infections in scars and wounds (Table 4). The greater efficacy of this compound against these strains finds its application against food and topical pathogenesis. Therefore, further subsequent drug safety and molecular action studies of this compound are an utmost need for its global use. The release of LDH enzyme with content with a concomitant gradual decrease in CFU value indicates that the compound affects cellular permeability and thus rendered a quick death of the cells^30,31^. Moreover, the compound is stable up to 100 °C, superior to the antifungal peptide that was stable up to 70 °C produced by *A. clavatus*^42^. Therefore, compared to the other antimicrobial compounds produced by *Aspergillus* spp., the present antimicrobial compound is more heat stable and could be used as antimicrobials in many processes involving thermal treatment up to 100 °C but not autoclaved processes.

The drug efficacy is nowadays being trialled with combination mode^43–45^. Very often, it was observed that co-administration of more than one active compound might enhance or reduce the drug efficacy of the lead compound^43–45^. Therefore, synergistic/antagonistic study is very much essential for drug potentization. The study revealed that the compound 5-butyl-2-pyridine carboxylic acid has a synergistic effect with three conventional antibiotics (Fig. 6d) like ciprofloxacin which acts on bacterial topoisomerase II (DNA gyrase) and topoisomerase IV, streptomycin that binds irreversibly to the 16S rRNA and S12 protein within the bacterial 30S ribosomal subunit, and vancomycin that inhibits bacterial cell-wall biosynthesis (https://go.drugbank.com/drugs/DB00512). Moreover, these combinations would reduce the application of the antibiotic dose to cure many challenging pathogens. The literature study suggested that the combined antimicrobial effect of antibiotics and extracted metabolites increase by increasing their bonding reaction^46–49^.

Many fungal secondary metabolites show important biological efficacies like antibacterial and antiviral activities mainly targeting different microbial proteins, like DNA-gyrase, topoisomerase IV, dihydrofolate reductase, transcriptional regulator TcaR (protein), and aminoglycoside nucleotidyltransferase. However, the metabolites acting on respiratory enzymes are very rare. The present study shows that the 5-butyl-2-pyridine carboxylic acid has a strong binding affinity (Supplementary Table S5) towards the quinol-fumarate reductase (QFR), succinate: quinone oxidoreductase (SQR, succinate dehydrogenase) and menaquinol: fumarate oxidoreductase (QFR, fumarate reductase), members of the integral membrane proteins Complex II family, that play a key role in the Krebs cycle. Hence, the molecular docking studies (Supplementary Fig. S5) revealed that the compound targetting QFR of *E. coli* inhibits the essential respiratory enzymes, thus leading to energy depletion and cellular viability. This observation is different from the novel anthraquinone, 2-(dimethoxymethyl)-1-hydroxyanthracene-9,10-dione, isolated from *A. versicolor,* that had efficacy against topoisomerase IV and AmpC β-lactamase enzymes^50^. Thus, molecular docking studies also revealed a novel target site of action of the 5-butyl-2-pyridine carboxylic acid that could be used as a future drug in combating many infectious and chronic diseases.

## Conclusions

The species of *Aspergillus* are the leading microfungi that have wide use in different industries. They produce a diverse array of potential biomolecules like antibacterial, antifungal, immunodepressants, anti-AIDS drugs, etc. However, reports on the broad-spectrum antimicrobials from this organism are very limiting. The development of new, novel and high potential antimicrobials is a global challenge due to the upsurge in multidrug resistance among the food and topical pathogens. To this critical demand, the present study reports for the first time that *A. fumigatus* nHF-01 produces a broad-spectrum 5-butyl-2-pyridine carboxylic acid antibacterial compound that has activity against human pathogenic bacteria on both the planktonic and biofilm states. It has absolute lethality at 15 h treatment at a dose of MICx2. Moreover, the compound has a strong binding affinity towards the respiratory enzymes resulting in rapid depletion of energy and subsequent death of cells. This robust broad-spectrum antibacterial compound could be produced in a very low-cost media. Further analysis with a detailed pharmacological mechanism of action study would decipher the antibacterial action more vividly and thus, be trialled as a potent drug contributing to human endeavour in future.

## Materials and methods

### Fungal strain and sensitivity towards antifungal drugs

The micro-fungus *A. fumigatus* nHF-01 (GenBank Acc. No. MN190286) was cultured and maintained in Potato Dextrose Agar (PDA) at 28 °C and sub-cultured in every 5–7 days interval^13^. The antifungal drug sensitivity of this strain was tested against many azole and systemic fungicide drugs (Supplementary Table S1) by agar well diffusion assay and MIC was determined following Mandal et al.^13^.

### Bacterial strains and the assessment of antibacterial efficacy

For the antimicrobial assay, the strain was grown in respective culture broth in a BOD incubator at 28 °C for 10 days. After profuse growth and sporulation, the culture aliquots were tested for their antibacterial efficacy by the agar well diffusion method on nutrient agar (NA) plates following the standard protocol^13,51^, against the human pathogenic Gram-positive and Gram-negative bacteria (Supplementary Table S2). The antibacterial potency was determined as inhibition zone diameter against the targeted bacteria, viz*. Bacillus cereus* MTCC 1272, *B. subtilis* MTCC 411, *Enterococcus faecalis* MCC 2041T, *Escherichia coli* MTCC 723, *E. coli* ATCC DH5α, *Klebsiella pneumonia* MTCC 7407*, Mycobacterium smegmatis* mc^2^ 155*, Pseudomonas aeruginosa* MTCC 741*, Salmonella enterica* serovar Typhimurium MTCC 98, *Staphylococcus aureus* MTCC 96, *S. epidermidis* MTCC 3086, *Streptococcus mutans* MTCC 890, and *Vibrio parahaemolyticus* MTCC 451. The experiment was repeated as triplicate trials. The ATCC, MCC and MTCC strains were procured from American Type Culture Collection, USA, Microbial Culture Collection, Pune, India and IMTECH, Chandigargh, India, and maintained in the media suggested by the repository houses. The bacterial stock cultures were maintained in 70% glycerol at − 20 °C and were sub-cultured twice before experimentation.

### Screening of media and submerged culture conditions for antibacterial compound production

To screen the effect of media constituents and the culture conditions on the AMC production, different standard media, viz. Malt Extract Broth (MEB; containing sprouted malt grains extract 2%, w/v), Czapekdox Broth (CZB, 35.01 g/L; containing sucrose 30 g/L, sodium nitrate 3 g/L, dipotassium phosphate 1.0 g/L, magnesium sulphate 0.50 g/L, potassium chloride 0.50 g/L and ferrous sulphate 0.01 g/L, w/v), Potato Dextrose Broth (PDB, 24 g/L; containing potato infusion 20%, dextrose 2%, w/v), Nutrient Broth (NB; 13 g/L; containing glucose 1 g/L, peptone 15 g/L, sodium chloride 6 g/L, yeast extract 3 g/L, w/v), and Corn Meal Broth (CMB; 17 g/L; containing cornmeal infusion 50 g/L, w/v) were used at different concentrations viz*.* 2%, 4%, 6%, 8% and 10% (w/v), set at different pH (3.0, 4.0, 5.0, 6.0, 8.0, and 10.0.) using 5(N) NaOH and 5(N) HCl in 100 mL batch culture and in shake condition at 100 rpm in a rotary shaker incubator (SNS, Kolkata, India). Five agar plugs containing the 5–7 days old mycelial culture plate (PDA) were inoculated in each flask and incubated at different temperatures, viz. 20 °C, 28 °C, 37 °C and 45 °C, in a BOD shaker incubator. The culture aliquots were taken out at different incubation periods, such as 5th, 7th, 10th, 15th, and 20th day, and cell-free culture aliquot was harvested by centrifugation at 7168×*g* for 10 min at 4 °C. The supernatant was fractionated with an equal volume of *n*-hexane, dichloromethane (DCM), and ethyl acetate solvent in a separating funnel; the solvent phase was harvested and evaporated in a rotary-vacuum evaporator (Superfit, Model: PBV-7D, Mumbai, India) and used for antibacterial activity assay. The fungal biomass, extractable compound and specific activity were also recorded. All the experiments were repeated three times.

### Optimization of culture conditions

After screening the primary media constituents influencing AMC production, two critical factors, i.e. Yeast extract and Malt extract, were tested in different proportions. Therefore, the batch fermentation (100 mL) were conducted containing different % ME (0 to 8, w/v) and % YE (0 to 8, w/v) set at pH 6.0, 20 °C for ten days in shake condition, and the amount of AMC production was recorded as described above. Approximately 1 L of batch fermentation was conducted to harvest an ample amount of AMC for purification and characterization studies.

### Chromatographic purification of the active compound

The analytical and preparative TLC (Thin layer chromatography) was performed using TLC silica gel 60 F_254_ plates (Merck, Germany). The compounds of the crude extract were separated in n-hexane and ethyl acetate solvent system (9:10, v/v), visualized under 254 nm UV light, and the *Rf* values were recorded. The potent antibacterial fraction was determined through TLC zymogram assay by overlaying NA soft-agar seeded with sensitive bacteria. The potent antibacterial spot was marked by observing the growth inhibition zone and tallied with the *Rf* values. Similarly, several preparative TLC plates were run, and the potent AMC was scraped out from the TLC plates, extracted with DCM, and evaporated to dryness. Preparative HPLC analysis of the separated fraction was carried out to harvest the pure compound in a multidimensional RP-HPLC system (Water Alliance, 2695, MDLC, UV–Vis detector 2487) equipped with a C_18_ column. Elution was done in a 0.5% H_3_PO_4_, 90% acetonitrile, and 0.1% trifluoroacetic acid as mobile phase with a flow rate of 1.0 mL/min at 30 ± 2 °C and the detected at 246 nm. The fractions were collected and tested for potential antibacterial activity, as mentioned above. Several runs were conducted to harvest the pure compound for further characterization.

### Characterization of the active compound

#### UV–Vis and FT–IR analysis

The UV–visible spectrum of the HPLC fraction was carried out in a UV–Vis spectrophotometer (UV–Vis 1800, Shimadzu, Japan) using DCM as a solvent system. For the FT-IR chromatogram (IR Infinity-1S, Shimadzu, Japan), 1 mg of the compound was dissolved in 500 µL DCM and placed for FT–IR measurement in the wavelength range 650 to 3050 cm^−1^ at 16 cm^−1^ resolution for 1374 scans.

#### GC–MS, ESI–MS and NMR analysis

To determine the putative AMC in the active fraction, gas chromatography-mass spectrometry (GC–MS) of TLC C4 fraction was performed in a GC–MS instrument (GC-240 Ion Trap MS, Model:7890B, Agilent Technologies, USA) equipped with a capillary column VF-5MS (Length 31.98 min, ID-0.25MM, Film-0.25 µm, Max temp. 325 °C). A scan rate of 3 micro-scans (1.44 s/scan) with a data rate of 0.69 Hz and mass detection range 0–680 *m/z* was set. The instrument was operated in electron impact mode at an emission current of 25 Amps, injector temperature of 250 °C, and detector temperature of 300 °C. The sample was loaded at an initial oven temperature of 50 °C (isothermal for 2 min), 7.6522 psi pressure, and a flow rate of 1 mL/min for 32 min run time. The identification of the compounds was made with the spectral data match with the NIST library. The mole percentage was calculated by the formula: Mole% = Ai/Ac × 100, where, Ai = peak area count of the individual compound, and Ac = cumulative peak area count of all compounds.

The ESI–MS data of the HPL fraction was obtained using the Xevo G2-XS QT ESI–MS instrument (Waters Zspray^TM^LackSpray) connected to a capillary column. The sample was dissolved in methanol and run in a positive ion mode in the mass range of 100–400 *m/z*. The ^1^H-NMR was carried out in the BrukerBioSpin instrument (Topspin v1.3, USA). 10 mg sample dissolved in 0.6 mL deuterated chloroform (CDCl_3_) solvent was loaded into a 5 mm Wilmad 528-PP NMR tube. NMR was run at: Operating temperature 28 °C; Proton spectra recorded at 64 K; Spectral width 10.3 ppm, D1 1 s, NS 51. The chemical shifts were recorded in parts per million (ppm, δ) and the coupling constants at 600 MHz.

### Phytochemical screening, solubility and thermo-stability studies

Chemical tests for active constituents of the pure compound were carried out following Mandal et al.^31^. The thermo-stability of the compound was checked at 60 °C, 80 °C, 100 °C in a water bath, and at 121 °C in an autoclave for 30 min. The residual antimicrobial activity was assayed against the above mentioned pathogenic bacteria by agar well diffusion assay. The solubility was checked in different organic solvents, viz. n-hexane, diethyl ether, DCM, ethyl acetate, methanol, DMSO and chloroform. The physical appearances and colour were also observed.

### Antimicrobial potentiality studies

#### Determination of MIC and MBC

Serial dilution in the range of 35.7 mg/mL to 0.069 mg/mL was prepared with DCM solvent and tested against the target bacteria. The MIC is the lowest concentration of the active extract treatment, where no visible growth is observed. 20 µL of different concentrations was applied by the agar well diffusion plates seeded with each bacterial strain at Mc Farland standard (0.6 at 610 nm), incubated at 37 °C for 48 h and observed for the zone of inhibition. The MBC was defined as the minimum concentration of the antibacterial compound) that produced no viable cells. The actively grown sensitive bacteria were treated with compounds higher than MIC values and incubated for 48 h. The number of viable cells was determined by counting the colony-forming units (CFU/mL) by decimal dilution plating^31^.

### Effect of the active fraction on growth and viability of bacterial strains

The effect of the AMC was studied on sensitive human pathogenic bacterial strains such as *B. cereus*, *E. coli, S. epidermidis,* and *S. enterica* serovar Typhimurium. The 50 µL of purified active compound (MIC × 2 dose) was added to the 210 µL of exponentially growing cells and incubated at 37 °C for 33 h. The growth was monitored at OD_620_ nm in a spectrophotometer (UV–Vis 1800, Shimadzu, Japan) and viability by the decimal diluted plate-count method. For the plate count method, the treated and control cells were serially decimal diluted and 500 µL of the dilution (10^–2^ to 10^–8^ dilution) were plated on NA plates and incubated at 37 °C. The viable colonies (CFU) were counted from respective treatment plates at 48 h and calculated as log CFU/mL. The values were compared with the standard antibiotics, viz. ciprofloxacin, streptomycin and vancomycin at MICx2 and MICx50 doses against the bacterial strains *B. cereus* and *E. coli*.

### Determination of time-kill kinetics and time-kill endpoint

The time-kill test was done with the test sample at a concentration of MBC of the bacterial strains such as *B. cereus*, *E. coli, S. epidermidis,* and *S. enterica* serovar Typhimurium. The actively grown bacterial strains (log-phase growth, at Mc Farland standard, 0.6) were treated with the pure compound and incubated at 37 °C for 30 h. At different times of incubation (0 to 30 h, 3 h interval), 100 µL of the treated culture was taken out and serially diluted at 10^–1^ to 10^–4^ time, spread on NA plates in triplicate, incubated at 37 °C for 48 h and observed for nos. of viable colony development. The viable counts were calculated as log CFU/mL, and kill curves were plotted against incubation time. A bactericidal effect is defined as a decrease in the logarithm value of the CFU/mL over a specified time^52^.

### Effect on cellular integrity and cell morphology

The active growing cells were treated with MIC × 2 dose of the bacterial strains such as *B. cereus*, *E. coli, S. epidermidis,* and *S. enterica* serovar Typhimurium, and the amount of LDH in the cell-free lysate was measured for 30 min, 60 min and 480 min^31^. The sonication (Power 95%, Gap 2s, Temp 20 °C, Stroke 10 times) was done in a Probe Sonicator (PKS-250FM, USA), and the untreated cell suspension was treated as positive and negative controls, respectively. The effect on cellular morphology was studied by scanning electron microscopy (SEM). Ten μl (MIC × 2) of the compound was mixed with a 100 μL of 6–8 h grown bacterial culture of *B. cereus* and *E. coli*, incubated at 37 °C for 3 h. The untreated and treated cells were washed with sterile sodium-phosphate buffer (100 mmol/L, pH 7.0) and were processed for SEM study (EVO 18, Zeiss, Oxford, England with Q150R ES, Quorum gold sputter) following Mandal et al.^31^.

### Antibiofilm and biofilm destabilization assay

Antibiofilm assay of the AMC was performed in microwell plate seeded with 95 µL of active cell suspension of *B. cereus* and *E. coli* (at Mc Farland standard (0.6), ~ 1 × 10^6^ cells/mL) in the nutrient broth and 5 µL of active compound at the MIC/4, MIC/2, and MIC concentration (4, 8, and 16 µg/mL, respectively, w/v) were added in wells. The experimental setup was incubated at 37 °C for 24–36 h under static conditions. Similarly, for biofilm destabilization assay the 18 h preformed biofilm of the strains were treated with active compound at the same concentration mentioned above and incubated at 37 °C for 24–36 h under static conditions. After incubation, the culture aliquote was decanted, and the wells were washed with 150 µL of distilled water to remove the planktonic cell and repeat twice. The adherent cells in wells were stained with 0.1% (w/v) Crystal violet solution (in water) to stain the polysaccharides of the biofilm and kept for 15 min at room temperature. The plate was gently drained upside down on tissue paper 3–4 times and allowed drying. 125 µL of 30% (v/v) acetic acid was added to the wells and incubated for 10–15 min. OD of stained adherent bacteria was determined with a microplate reader (Microplate reader, Bio-Rad, iMark™, USA) at a wavelength of 595 nm. Usnic acid (4, 8, and 16 µg/mL, w/v) was used as a positive control and culture only with the media served as a negative control^53^. The per cent of biofilm inhibition was evaluated using the following formula. % of biofilm inhibition = 100 × [(Control OD_595_ nm − Test OD_595_ nm)/Control OD_570_ nm]^54^.

### Cytotoxicity assay

MTT (3-[4,5-dimethylthiazol-2-yl]-2,5-diphenyltetrazolium bromide) based cytotoxicity assay was done on A549 (ATCC number CCL-185) mammalian alveolar cancer cell line maintained in Dulbecco Modified Eagle Medium (DMEM) supplemented with 10% fetal bovine serum with 1% Penicillin–Streptomycin at 37 °C in an incubator with 5% CO_2_. After one hour, the absorbances were measured at 590 nm (Microplate reader, Bio-Rad, iMark™, USA)^55^. All tests were performed in triplicate.

### Evaluation of synergistic/antagonistic effects between AMC and antibiotics

The synergy between two antimicrobial compounds is often expressed in terms of the fractional inhibitory concentration (FIC) which is expressed by the MIC of the compound in combination divided by the MIC of antibiotic acting alone. To determine the FIC, the target organisms *B. cereus* and *E. coli* were fresh cultured, harvested, and suspended in sterile NB to produce a McFarland value of 0.5 (OD_610 nm_). The suspension was diluted in fresh NB to achieve a final CFU of 4 × 10^6^ to 5 × 10^6^ from which 10 µL was inoculated into the microwell plates and incubated at 37 °C for 16–20 h*.* A checkerboard microdilution technique was used to examine the synergism between the antibiotics and AMC against test organisms. All tests were carried out in a duplicated manner. The values were inferred based on the nature of the interaction like FICI < 0.5 = synergy, 0.5 ≤ FICI < 1 = partial synergy, FICI = 1 additive, 2 ≤ FICI < 4 = indifferent, and > 4 FICI = antagonism^56^. FICI was calculated as follows: FIC of AMC = MIC of AMC in combination/MIC of AMC alone^57^. The calculated FIC index was used to detect the nature of the interaction between the two test agents and the interaction either synergism or indifference or antagonism type.

### Molecular modelling

The crystal structure of many AMC target proteins/enzymes were obtained from a protein data bank (http://www.rcsb.org). The structures were then cleaned using Autodock tools by removing heteroatoms and by adding necessary hydrogen atoms. The structure of the 5-butyl-2-pyridine carboxylic acid molecule was obtained from PubChem (https://pubchem.ncbi.nlm.nih.gov/). Using UCSF Chimera^58^, the PDB files of the 5-butyl-2-pyridine carboxylic acid were created for docking. Autodock Vina^59^ package was used for docking between the best binding sites of enzyme and proteins and 5-butyl-2-pyridine carboxylic acid. All the docked compounds were subjected to further selection for ADMET property analysis based on Lipinski's five (Ro5) rule, and compounds with any Ro5 violations were eliminated. Ro5 includes molecular weight, lipophilicity, molar refractivity, number of hydrogen bond donors and acceptors. The Molinspiration server was used for calculating the physicochemical properties of compounds (http://www.molinspiration.com/cgi-bin/properties).

### Statistical analysis

Exploratory analysis was done to find the best media composition^60,61^. Bar plots and a heatmap clustered based on similar performance were used to understand better the effects of media composition on biomass, extractable compound and specific activity. One-way ANOVA was used to analyse the variation between experimental sets. The correlation matrix was used for understanding the correlation between parameters. Response Surface Modeling (RSM) was used to determine the interaction between parameters^62^. The concentration of MEB and YE were treated as a predictor variable, biomass, extractable compound and specific activity were treated as the response variable for creating the RSM model; details of the experimental design and results were given in Table 2. We created three models for three response variables, including all the first order, second-order, and interaction terms; details of the model and coefficients are given in Table 3. Predictions were made from the model and compared with observed variables. Clustering and production of heatmap were done using Orange 3 (https://orangedatamining.com/) software. The rest of the analysis, modelling and plotting were done using R 3.6.3 (Cran.r-Project.Org) software. All the codes of R were run in Rstudio environment 1.2.5042 (www.rstudio.com).

## Supplementary Information

Supplementary Information.

## Data Availability

The datasets generated during and/or analysed during the current study are available from the corresponding author on reasonable request.

## Code availability

*Database Accession Numbers*: The GenBank Accession number of *Aspergillus fumigatus* nHF-01 is MN190286.

## References

[1] Hassan SAA, Bakhiet SEA (2017). Optimization of antibacterial compounds production by *Aspergillus fumigatus* isolated from sudanese indigenous soil. Int. Biol. Biomed. J..

[2] Smith, D. & Ryan, M. J. *Fungal Sources for New Drug Discovery* 131–133 (McGraw-Hill Yearbook of Science & Technology, 2009).

[3] Aly HA, Debbab A, Proksch (2011). Fungal endophytes: Unique plant inhabitants with great promises. Appl. Microbiol. Biotechnol..

[4] Greco C, Keller NP, Rokas A (2019). Unearthing fungal chemodiversity and prospects for drug discovery. Curr. Opin. Microbiol..

[5] Furtado NAJC, Said S, Yoko Ito IY, Bastos JK (2002). The antimicrobial activity of *Aspergillus fumigatus* is enhanced by a pool of bacteria. Microbiol. Res..

[6] Mikawlrawng, K. *Aspergillus in Biomedical Research. New and Future Developments in Microbial Biotechnology and Bioengineering* 229–242 (Elsevier, 2016). 10.1016/B978-0-444-63505-1.00019-1.

[7] Denning DW, Anderson MJ, Turner G, Latgé JP, Bennett JW (2002). Sequencing the *Aspergillus fumigatus* genome. Lancet Infect. Dis..

[8] Bennett JW (2009). *Aspergillus*: A primer for the novice. Med. Mycol..

[9] Romsdahl J, Wang CCC (2019). Recent advances in the genome mining of *Aspergillus* secondary metabolites (covering 2012–2018). Med. Chem. Commum..

[10] Debeaupuis JP, Sarfati J, Chazalet V, Latge' JP (1997). Genetic diversity among clinical and environmental isolates of *Aspergillus fumigatus*. Infect. Immun..

[11] Rochfort S (2005). A novel aspochalasin with HIV-1 integrase inhibitory activity from *Aspergillus flavipes*. J. Antibiot..

[12] Jain P, Pundir RK (2011). Effect of fermentation medium, pH and temperature variations on antibacterial soil fungal metabolite production. J. Agric. Technol..

[13] Mandal V, Adhikary R, Maiti PK, Mandal S, Mandal V (2021). Morpho-biochemical and molecular characterization of two new strains of *Aspergillus fumigatus* nHF-01 and *A. fumigatus* PPR-01 producing broad-spectrum antimicrobial compounds. Braz. J. Microbiol..

[14] Lubertozzi D, Keasling JD (2009). Developing *Aspergillus* as a host for heterologous expression. Biotechnol. Adv..

[15] Luyen ND (2019). Aspermicrones A-C, novel dibenzospiroketals from the seaweed-derived endophytic fungus *Aspergillus micronesiensis*. J. Antibiot..

[16] Kaur N, Arora DS, Kalia N, Kaur M (2020). Antibioflm, antiproliferative, antioxidant and antimutagenic activities of an endophytic fungus *Aspergillus fumigatus* from *Moringa oleifera*. Mol. Biol. Rep..

[17] Limbadri S (2018). Bioactive novel indole alkaloids and steroids from deep sea-derived fungus *Aspergillus fumigatus* SCSIO 41012. Molecules.

[18] El-Sayed ASA (2020). Production and bioprocess optimization of antitumor Epothilone B analogue from *Aspergillus fumigatus*, endophyte of *Catharanthus roseus*, with response surface methodology. Enzyme Microb. Technol..

[19] El-Sayed E-SR, Ahmed AS, Hassan IA, Ismaiel AA, Karam El-Din A-ZA (2019). Strain improvement and immobilization technique for enhanced production of the anticancer drug paclitaxel by *Aspergillus fumigatus* and *Alternaria tenuissima*. Appl. Microbiol. Biotechnol..

[20] Liu JY (2004). *Aspergillus fumigatus* CY018, an endophytic fungus in *Cynodon dactylon* as a versatile producer of new and bioactive metabolites. J. Biotechnol..

[21] Wang W (2018). Secondary metabolites isolated from the deep sea-derived fungus *Aspergillus sydowii* C1–S01-A7. Nat. Prod. Res..

[22] Wang W (2017). Cytotoxic and antibacterial compounds from the coral-derived fungus *Aspergillus tritici SP2-8-1*. Mar. Drugs.

[23] Wang Y, Zheng J, Liu P, Wang W, Zhu W (2011). three new compounds from *Aspergillus terreus* PT06-2 grown in a high salt medium. Mar. Drugs.

[24] Zheng J (2013). Antimicrobial ergosteroids and pyrrole derivatives from halotolerant *Aspergillus flocculosus* PT05-1 cultured in a hypersaline medium. Extremophiles.

[25] Kollakalnaduvil Raghavan KRM (2020). Characterisation of an extracellular thermo stable antibacterial peptide from marine fungus with biofilm eradication potential. J. Pharm. Biomed. Anal..

[26] Othman AM, Elsayed MA, Al-Balakocy NG, Hassan MM, Elshafei AM (2019). Biosynthesis and characterization of silver nanoparticles induced by fungal proteins and its application in different biological activities. J. Genet. Eng. Biotechnol..

[27] Alavi M, Karimi N (2017). Characterization, antibacterial, total antioxidant, scavenging, reducing power and ion chelating activities of green synthesized silver, copper and titanium dioxide nanoparticles using *Artemisia haussknechtii* leaf extract. Artif. Cells Nanomed. Biotechnol..

[28] Burmeister HR, Grove MD, Peterson RE, Weisleder D, Plattner RD (1985). Isolation and characterization of two new fusaric acid analogues from *Fusarium moniliforme* NRRL 13,163. Appl. Environ. Microbiol..

[29] Cushnie TPT, Cushnie B, Lamb AJ (2014). Alkaloids: An overview of their antibacterial, antibiotic-enhancing and antivirulence activities. Int. J. Antimicrob. Agents.

[30] Mandal V, Sen SK, Mandal NC (2010). Assessment of antibacterial activities of pediocin produced by *Pediococcus acidilactici* Lab 5. J. Food Saf..

[31] Mandal M (2016). In vitro antibacterial potential of *Hydrocotyle javanica* Thunb. Asian Pac. J. Trop. Dis..

[32] Dagenais TRT, Keller NP (2009). Pathogenesis of *Aspergillus fumigatus* in invasive aspergillosis. Clin. Microbiol. Rev..

[33] Kiran GS (2009). Optimization and production of a biosurfactant from the sponge-associated marine fungus *Aspergillus ustus* MSF3. Colloids Surf. B.

[34] Barakat KM, Yousry MG (2012). Antimicrobial agents produced by marine *Aspergillus tereus* Var. Africanus against some virulent fish pathogens. Indian J. Microbiol..

[35] Compaore H (2016). Optimization of antimicrobial compound production by *Aspergillus fumigatus* isolated from maize in Ouagadougou, Burkina Faso. Curr. Res. Microbiol. Biotechnol..

[36] Agastian P, Merlin JN, Christhudas IVSN, Kumar P (2013). Optimization of growth and bioactive metabolite production: *Fusarium solani*. Asian J. Pharm. Clin. Res..

[37] Kang D (2013). Culture condition-dependent metabolite profiling of *Aspergillus fumigatus* with antifungal activity. Fungal Biol..

[38] Thanh TT, Quoc TN, Xuan HL (2020). Fusaric acid and derivatives as novel antimicrobial agents. Med. Chem. Res..

[39] Sakagami Y (1999). Inhibitory activities of 2-pyridinecarboxylic acid analogs on phytogrowth and enzymes. Biol. Pharm..

[40] Nagasaka A (1985). Effect of fusaric acid (a dopamine β-hydroxylase inhibitor) on phaeochromocytoma. Clin. Endocrinol..

[41] Voss KA, Porter JK, Bacon CW, Meredith FI, Norred WP (1999). Fusaric acid and modification of the subchronic toxicity to rats of fumonisins in *F. moniliforme* culture material. Food Chem. Toxicol..

[42] Gargouri HS, Gargouri A (2008). First isolation of a novel thermostable antifungal peptide secreted by *Aspergillus clavatus*. Peptides.

[43] Rybak MJ, McGrath BJ (1996). Combination antimicrobial therapy for bacterial infections. Drugs.

[44] Misra D (2022). Anti-enteric efficacy and mode of action of tridecanoic acid methyl ester isolated from *Monochoria hastata* (L.) Solms leaf. Braz. J. Microbiol..

[45] Olajuyigbe OO, Afolayan AJ (2012). Synergistic interactions of methanolic extract of *Acacia mearnsii* De Wild. with antibiotics against bacteria of clinical relevance. Int. J. Mol. Sci..

[46] Jung WK (2008). Antibacterial activity and mechanism of action of the silver ion in *Staphylococcus aureus* and *Escherichia coli*. Appl. Environ. Microbiol..

[47] Birla SS (2009). Fabrication of silver nanoparticles by *Phoma glomerata* and its combined effect against *Escherichia coli, Pseudomonas aeruginosa* and *Staphylococcus aureus*. Lett. Appl. Microbiol..

[48] Fayaz AM (2010). Biogenic synthesis of silver nanoparticles and their synergistic effect with antibiotics: A study against Gram-positive and Gram-negative bacteria. J. Nanomed. Nanotechnol..

[49] Li WR (2011). Antibacterial effect of silver nanoparticles on *Staphylococcus aureus*. Biometals.

[50] Wang W (2018). Secondary metabolites isolated from the deep sea-derived fungus *Aspergillus sydowii* C1-S01-A7. Nat. Prod. Res..

[51] Dey BC, Rai N, Das S, Mandal S, Mandal V (2019). Partial purification, characterization and mode of action of bacteriocins produced by three strains of *Pediococcus* sp. J. Food Sci. Technol..

[52] May J, Chan CH, King A, Williams L, French GL (2000). Time-kill studies of tea tree oils on clinical isolates. J. Antimicrob. Chemother..

[53] Meenambiga SS, Rajagopal K (2018). Antibiofilm activity and molecular docking studies of bioactive secondary metabolites from endophytic fungus *Aspergillus nidulans* on oral *Candida albicans*. J. Appl. Pharm. Sci..

[54] Dawande AY, Gajbhiye ND, Charde VN, Banginwar YS (2019). Assessment of endophytic fungal isolates for its Antibiofilm activity on *Pseudomonas aeruginosa*. Int. J. Sci. Res. Biol. Sci..

[55] Maiti PK, Das S, Sahoo P, Sukhendu M (2020). *Streptomyces* sp SM01 isolated from Indian soil produces a novel antibiotic picolinamycin effective against multidrug-resistant bacterial strains. Sci. Rep..

[56] Botelho MG (2000). Fractional inhibitory concentration index of combinations of antibacterial agents against cariogenic organisms. J. Dent..

[57] Barapatre A, Aadil KR, Jha H (2016). Synergistic antibacterial and antibiofilm activity of silver nanoparticles biosynthesized by lignin-degrading fungus. Bioresour. Bioprocess..

[58] Pettersen EF (2004). UCSF Chimera—A visualization system for exploratory research and analysis. J. Comput. Chem..

[59] Trott O, Olson AJ (2010). AutoDock Vina: Improving the speed and accuracy of docking with a new scoring function, efficient optimization, and multithreading. J. Comput. Chem..

[60] Peng, R. D. Exploratory Data Analysis with R. Bookdown.Org. https://bookdown.org/rdpeng/exdata. (2020). Accessed from 10 Aug to 2 Dec 2021.

[61] ggplot2.barplot: Easy bar graphs in R software using ggplot2 - Easy Guides-Wiki-STHDA. (n.d.). www.Sthda.com. http://www.sthda.com/english/wiki/ggplot2-barplot-easy-bar-graphs-in-r-software-using-ggplot2. Accessed from 10 Aug to 2 Dec 2021.

[62] Lenth RV (2009). Response-surface methods in R, using rsm. J. Stat. Softw..

