# Supplementary material for: Production and characterization of a broad-spectrum antimicrobial 5-butyl-2-pyridine carboxylic acid from Aspergillus fumigatus nHF-01
Source: Sci Rep. 2022 Apr 9;12:6006. doi: 10.1038/s41598-022-09925-z (PMC8994762; doi:10.1038/s41598-022-09925-z)
Supplement: Supplementary file 1 — Supplementary Information. [file 41598_2022_9925_MOESM1_ESM.docx]

**Title: Production and Characterization of a Board-spectrum Antimicrobial 5-butyl-2-pyridine carboxylic acid from *Aspergillus fumigatus* nHF-01**

**Supplementary files:**

**
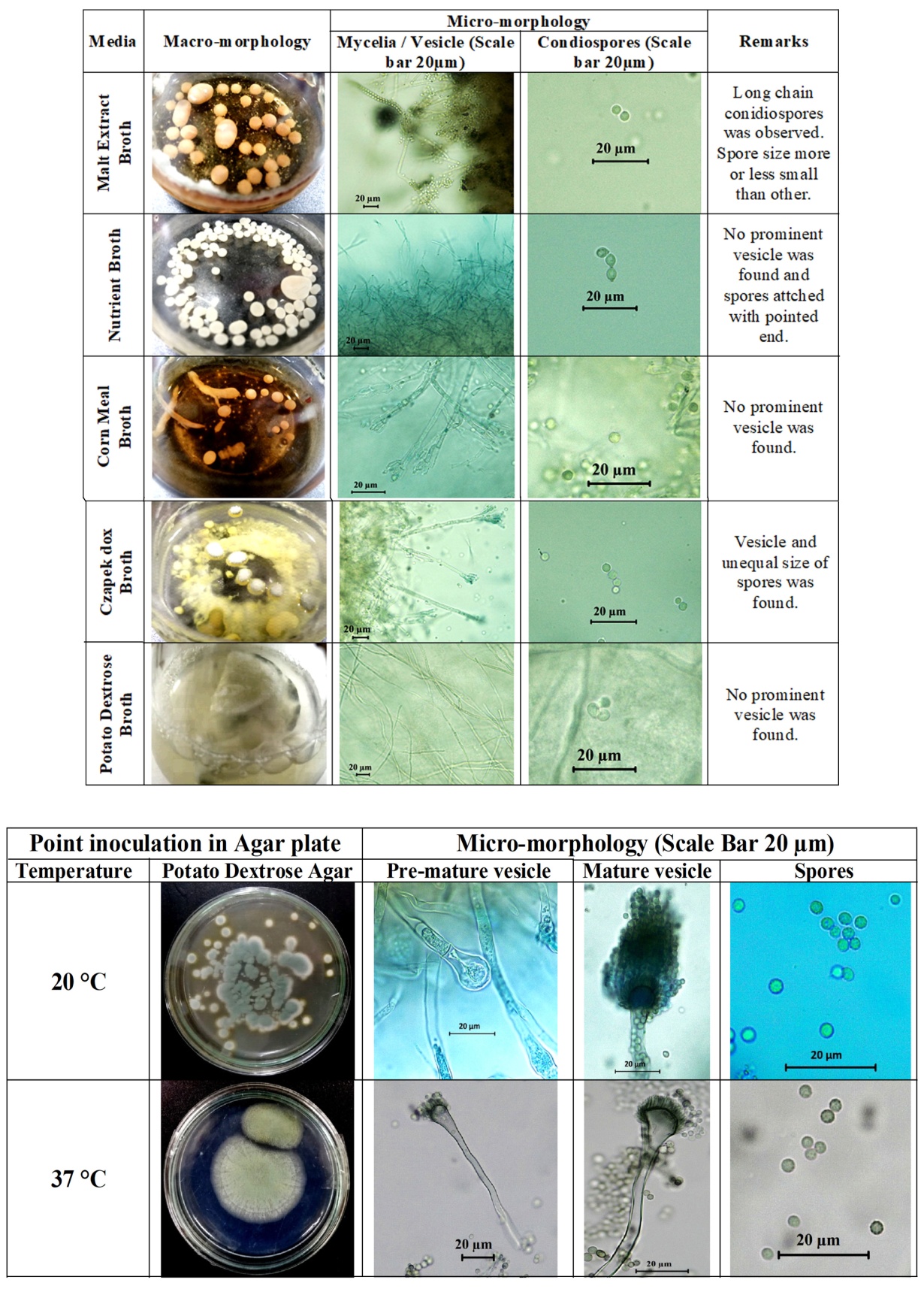
**

**Figure S1: The strain*Aspergillus fumigatus* nHF-01 and its macro and micro-morphology.** The above panel shows the shake flask broth culture, and the below panel shows the solid plate culture of the strain.


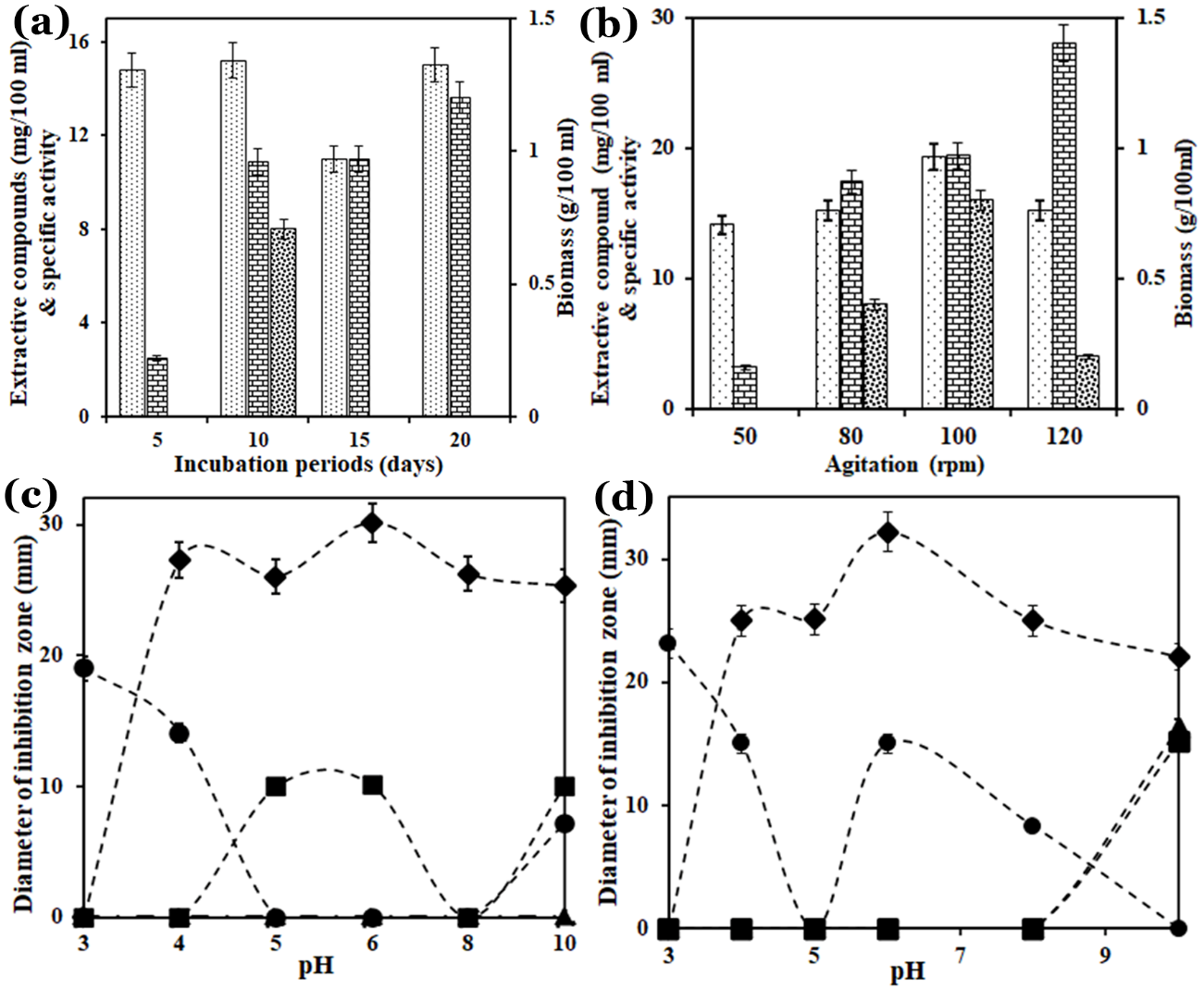


**Figure S2: Standardizing the optimum days, agitation, temperature and pH for antibacterial compound production. (a)** **optimum days; (b) optimum days, agitation; (c and d)** **optimum temperature and pH.** The figure indicates the assay of compounds extracted from different temperatures and pHs against Gram-positive bacterium *B. cereus* and Gram-negative bacterium *E. coli.* The rhomboid dotted line indicates 20 ºC, the round dotted line 28 ºC, the triangle dotted line 37 ºC, and the square dotted line indicates 45 ºC.


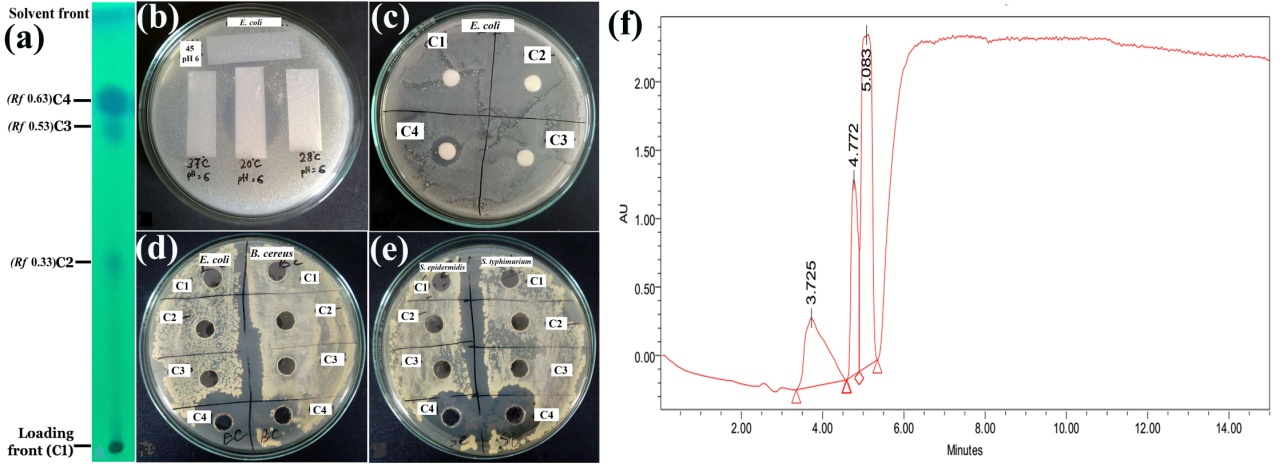


**Figure S3:** **Chromatographic analysis of the active extract. (a)** TLC of crude DCM extract; **(b)** TLC plate zymogram assay; **(c)** Paper disk assay of TLC scraps; **(d-e)** Agar-well diffusion assay of TLC scraps against the bacteria strain; and **(f)** HPLC of active C4 fraction. Here, TLC strips in fig. **'b'** indicate the TLC of active fraction from different pH and temperature levels.


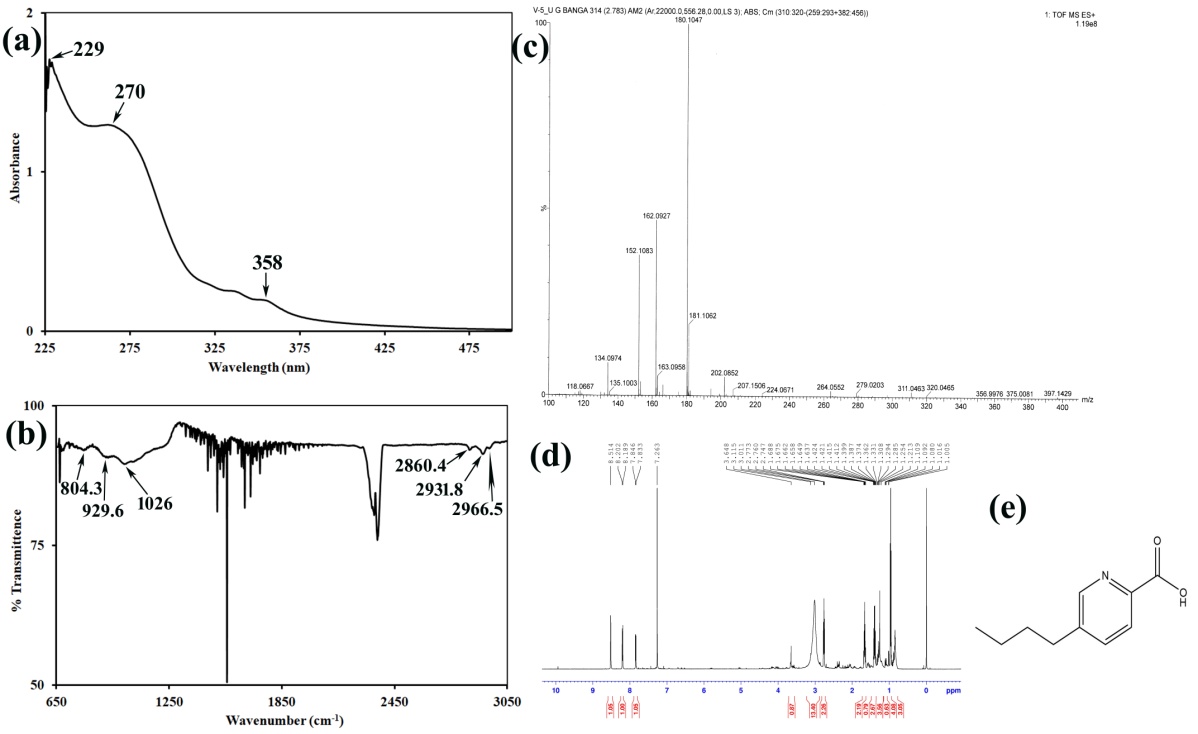


**Figure S4: Spectral analysis of pure compound of *A. fumigatus* nHF-01. (a)** UV-Vis spectrum; **(b)** FT-IR spectrum, **(c)** ESI-MS spectra, **(d)** ^1^H NMR; and **(e)** Chemical structure of 5-butyl-2-pyridine carboxylic acid.


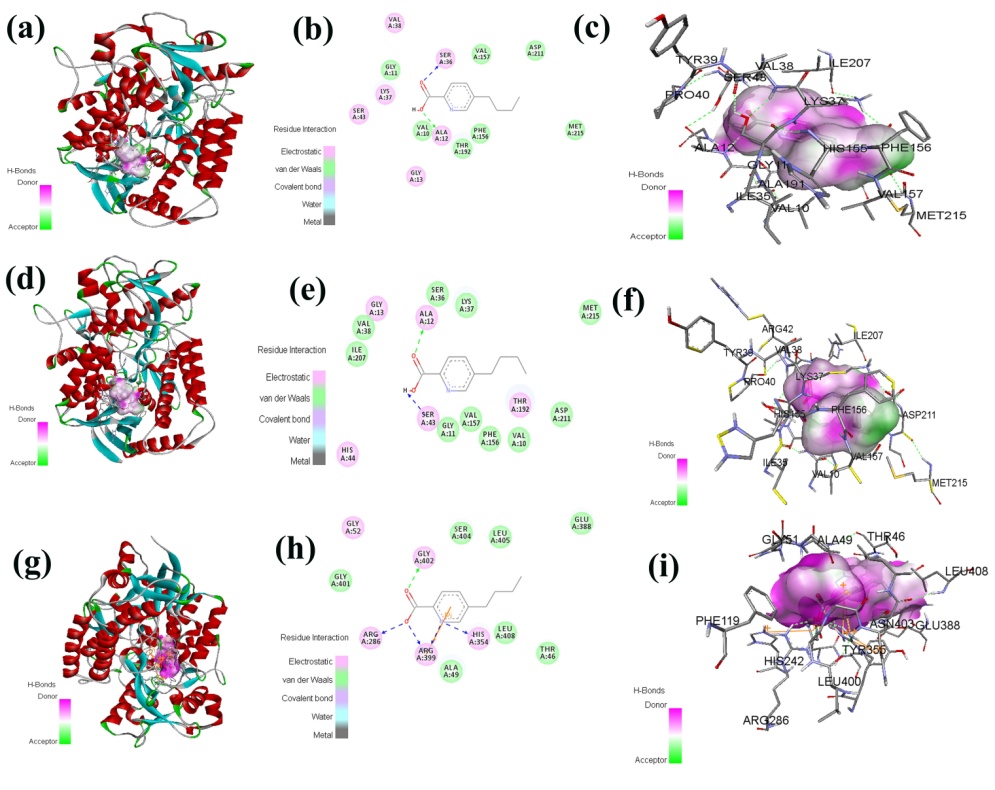


**Figure S5:** Docked confirmations of the active compounds of *A. fumigatus* nHF-01 with *E. coli* Quinol-Fumarate Reductase with Bound Inhibitor HQNO (PDB id- 1kf6; **Fig. a-c**), Quinol-Fumarate Reductase with Menaquinol Molecules (PDB id- 1l0v; **Fig. d-f)**, and *E. coli* succinate: quinone oxidoreductase (SQR) SdhB His207Thr (PDB id- 2wp9 **Fig. g-i**).


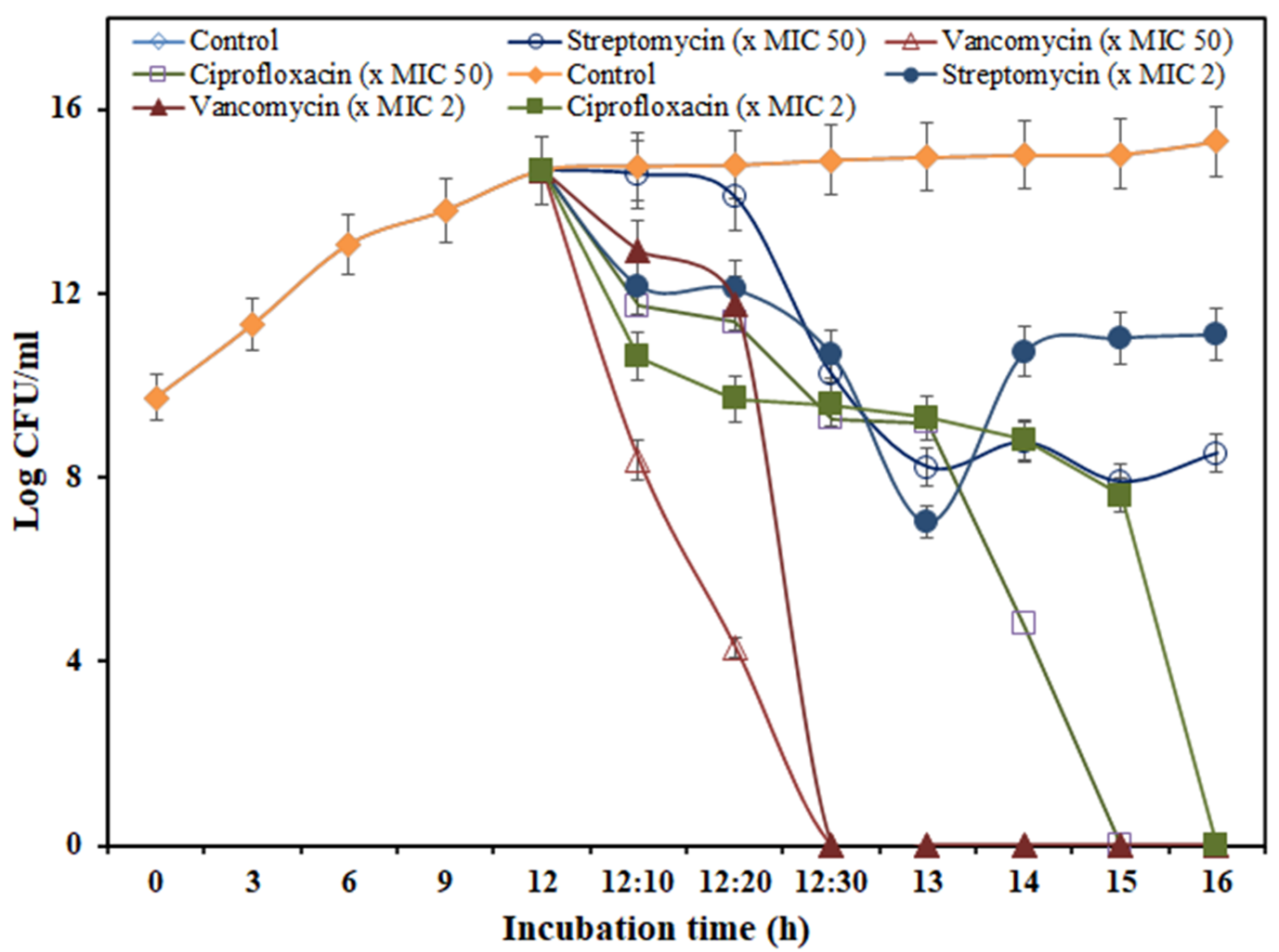


**Figure S6:** Effect of different antibiotics on *B. cereus* viability.

**Table S1: Sensitivity of 7 different antifungal drugs against *A. fumigatus* nHF-01**

| 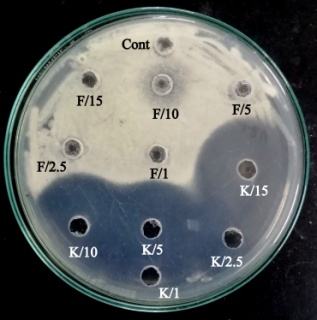 | 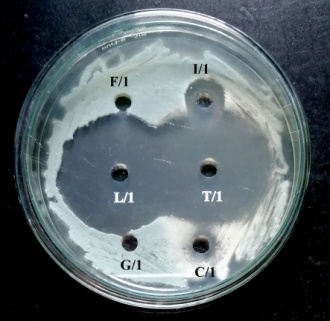 | 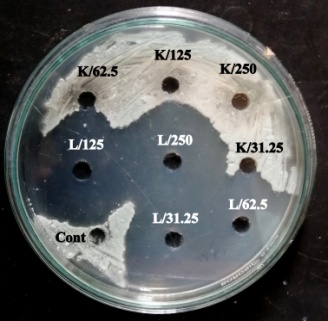 |
| --- | --- | --- |
| (Conc. 15-1 mg/ml; 20 µl) | (Conc. 1 mg/ml; 15 µl) | (Conc. 250-31.25 µg/ml; 12 µl) |
| 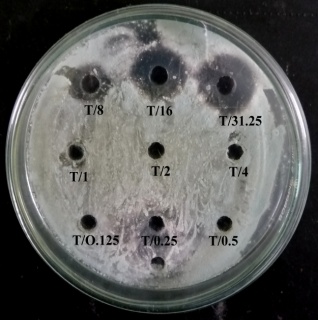 | 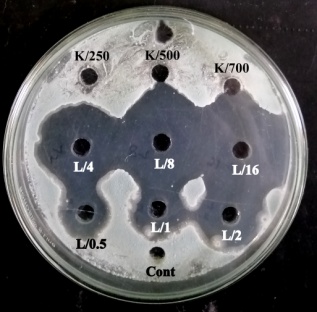 | 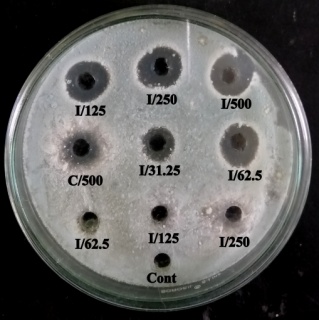 |
| (Conc. 31.25-0.125 µg/ml; 12 µl) | (Conc. 700-250 µg/ml & 16-0.5 µg/ml; 12 µl) | (Conc. 500-125 µg/ml & 500-62.5 µg/ml; 12 µl) |

**Abbreviation**: ‘**F’**= Fluconazole; ‘**K’**= Ketoconazole; ‘**L’**= Luliconazole; ‘**I’**= Itraconazole; ‘**G’**= Gresiofulvin; ‘**C’**= Clotrimazole; ‘**T’**= Terbinafine

**Table-S2: Antibacterial spectrum of the *A. fumigatus* nHF-01 compound in different media and solvents as evidenced by the diameter of growth inhibition zones.**

| **Culture media** |  |  |  | **Inhibition zone (35.7 mg/ml,mm) against** | | | | | | | | |
| --- | --- | --- | --- | --- | --- | --- | --- | --- | --- | --- | --- | --- |
|  |  |  |  | **Gram-negative strains** | | | | | **Gram-positive strains** | | | |
|  | **Biomass (g/100 ml)** | **Extractable compound (mg/100 ml)** | **Extraction Solvents** | ***E. coli*** | ***S. typhimurium*** | ***K. pneumonia*** | ***P. aeruginosa*** | ***S. epidermidis*** | | ***B. cereus*** | ***B. subtilis*** | ***E. faecalis*** |
| CMB | 0.24 | 42 | **DCM** | 15.14 ± 0.76 | 11 ± 0.55 | 11 ± 0.55 | 10 ± 0.5 | 14.11 ± 0.7 | | 00 | 07.2 ± 0.36 | 11 ± 0.55 |
| CZB | 0.72 | 68.4 |  | 17.17 ± 0.86 | 15.13 ± 0.75 | 11 ± 0.55 | 00 | 16 ± 0.8 | | 11 ± 0.55 | 07.8 ± 0.39 | 14 ± 0.7 |
| **MEB** | 0.96 | 68.8 |  | **22 ± 0.975** | **16 ± 0.8** | **16 ± 0.8** | **18.3 ± 0.91** | **17.21 ± 0.86** | | **19.2 ± 0.96** | **19.5 ±0.97** | **20 ± 0.1** |
| NB | 0.12 | 46.4 |  | 09.17 ± 0.42 | 08.2 ± 0.4 | 00 | 05 ± 0.25 | 00 | | 00 | 08 ± 0.45 | 00 |
| PDB | 0.16 | 38 |  | 00 | 09.1 ± 0.45 | 08.2 ± 0.41 | 00 | 09.11 ± 0.45 | | 09 ± 0.45 | 12 ± 0.6 | 00 |
| CMB | 0.24 | 34.1 | **Ethyl acetate** | 09 ± 0.45 | 00 | 11 ± 0.55 | 09 ± 0.45 | 00 | | 07.1 ± 0.35 | 09.1 ± 0.45 | 00 |
| CZB | 0.72 | 56.8 |  | 00 | 09.07 ± 0.4 | 00 | 00 | 00 | | 08.1 ± 0.40 | 11 ± 0.55 | 00 |
| MEB | 0.96 | 62 |  | 11 ± 0.55 | 09.1 ± 0.45 | 09 ± 0.45 | 10.2 ± 0.51 | 07.1 ± 0.32 | | 07 ± 0.33 | 07.8 ± 0.39 | 10.2 ± 0.5 |
| NB | 0.12 | 43.8 |  | 00 | 00 | 00 | 07 ± 0.35 | 08 ± 0.4 | | 08 ± 0.4 | 00 | 00 |
| PDB | 0.16 | 21.9 |  | 07.2 ± 0.36 | 01.12 ± 0.05 | 07.1 ± 0.35 | 07.8 ± 0.39 | 08 ± 0.40 | | 09 ± 0.45 | 07.1 ± 0.35 | 11 ± 0.55 |
| CMB | 0.24 | 41 | **n-hexane** | 00 | 08 ± 0.45 | 08 ± 0.4 | 10 ± 0.5 | 08.26 ± 0.41 | | 00 | 09 ± 0.45 | 05 ± 0.25 |
| CZB | 0.72 | 65.2 |  | 16.1 ± 0.805 | 00 | 00 | 11 ± 0.55 | 16.1 ± 0.805 | | 00 | 10 ± 0.5 | 00 |
| MEB | 0.96 | 66.7 |  | 14 ± 0.68 | 12.06 ± 0.6 | 12 ± 0.6 | 10 ± 0.5 | 14.02 ± 0.7 | | 11.1 ± 0.55 | 11 ± 0.55 | 15 ± 0.75 |
| NB | 0.12 | 38.9 |  | 09 ± 0.41 | 00 | 00 | 05 ± 0.25 | 00 | | 00 | 00 | 00 |
| PDB | 0.16 | 39.12 |  | 00 | 00 | 05 ± 0.25 | 00 | 00 | | 00 | 00 | 00 |

**Note:** Here, the number indicates the diameter of the inhibition zone (mm), and '00' indicates no inhibition zone. The values are the average of triplicate trials ± Standard error. Malt Extract Broth (MEB), Czapekdox Broth (CZB), Potato Dextrose Broth (PDB), Nutrient Broth (NB) and Corn Meal Broth (CMB) were the media used.

**Table-S3: GC-MS profiling of active compound of *Aspergillus fumigatus* nHF-01:**

| **Sl. No.** | **Chemical name** | **Peak number** | **Molecular formula** | **Retention time (min)** | **Molecular weight (g/mol)** | **Molecular structure** | **Mole %** |
| --- | --- | --- | --- | --- | --- | --- | --- |
|  | Dimethyl trisulfide | 1 | C_2_H_6_S_3_ | 7.689 | 126.25 |  | 0.201 |
|  | Pyridine, 3-butyl | 2 | C_9_H_11_Cl_2_N | 11.365 | 135.21 |  | 0.843 |
|  | Methanol, oxo-, benzoate | 3 | C_8_H_6_O_3_ | 11.476 | 168.15 |  | 0.148 |
|  | Tetrasulfide, dimethyl | 4 | C_2_H_6_S_4_ | 12.639 | 158.31 |  | 0.236 |
|  | Benzoic acid, 3,4-dimethyl-, methyl ester | 5 | C_10_H_12_O_2_ | 14.186 | 164.204 |  | 0.146 |
|  | Phenol, 2,4-bis(1,1-dimethylethyl)- | 6 | C_14_H_22_O | 17.319 | 206.329 |  | 0.49 |
|  | Gamma-Lumicolchicine | 7 | C_22_H_25_NO_6_ | 18.024 | 399.443 | 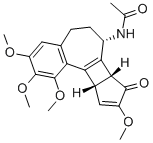 | 1.175 |
|  | Diethyl Phthalate | 8 | C_6_H_4_(COOC_2_H_5_)_2_ | 18.584 | 222.24 |  | 0.147 |
|  | **2-Pyridinecarboxylic acid, 5-butyl** | **9** | **C_10_H_13_NO_4_S** | **18.996** | **179.22** | **** | **5.15** |
|  | o-Anisic acid, 4-nitrophenyl ester | 10 | C_14_H_11_NO_5_ | 19.056 | 273.244 |  | 0.023 |
|  | 2'-Hydroxy-5'-methoxyacetophenone, tert-butyldimet | 11 | C_15_H_22_O_3_ | 19.886 | 250.16 |  | 0.075 |
|  | Nonadecane | 12 | C_19_H_40_ | 20.069 | 268.529 |  | 0.143 |
|  | Tetracontane, 3,5,24-trimethyl | 13 | C_43_H_88_ | 20.673 | 605.177 |  | 0.16 |
|  | Estra-1,3,5(10)-trien-17-one, 3,4-bis[(trimethylsilyl)oxy] | 14 | [C_24_H_38_O_3_Si_2_](https://pubchem.ncbi.nlm.nih.gov/#query=C24H38O3Si2) | 20.806 | 430.24 |  | 0.833 |
|  | Silane, dimethyl(2-methylphenoxy)docosyl | 15 | C_31_H_58_O_2_Si | 20.956 | 490.8765 |  | 4.65 |
|  | Tetratriacontyl heptafluorobutyrate | 16 | C_38_H_69_F_7_O_2_ | 21.541 | 690.94 |  | 0.232 |
|  | Isopropyl myristate | 17 | C_17_H_34_O_2_ | 21.762 | 270.457 |  | 0.211 |
|  | L-Proline, N-valeryl-, decyl ester | 18 | C_20_H_37_NO_3_ | 21.873 | 339.52 |  | 0.288 |
|  | 7-(1,3-Dimethylbuta-1,3-dienyl)-1,6,6-trimethyl-3, | 19 | [C_15_H_22_O_2_](https://pubchem.ncbi.nlm.nih.gov/#query=C15H22O2) | 21.922 | 236.17 |  | 0.316 |
|  | Propenamide, N-(2-bromo-4,6-difluorophenyl)-3-(2-m | 20 | C_9_H_6_BrF_2_NO | 22.681 | 262.019 |  | 0.373 |
|  | Hydroxylamine, O-decyl | 21 | C_10_H_23_NO | 22.862 | 173.3 |  | 1.237 |
|  | **No match** | **22** |  | **22.964** |  |  | **7.04** |
|  | Pyrrolo[1,2-a]pyrazine-1,4-dione, hexahydro-3-(2-m | 23, 25 | C_11_H_18_N_2_O_2_ | 23.118 | 210.2728 | 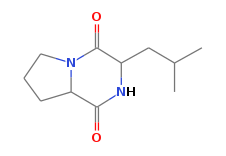 | 1.49 |
|  | Benzenepropanoic acid, 3,5-bis(1,1-dimethylethyl)- | 24 | C_17_H_26_O_2_ | 23.207 | 262.39 |  | 0.164 |
|  | Oxalic acid, isohexyl tetradecyl ester | 26 | C_22_H_42_O_4_ | 23.413 | 370.56648 |  | 0.535 |
|  | n-Hexadecanoic acid | 27 | C_16_H_32_O_2_ | 23.620 | 256.4241 |  | 2.282 |
|  | 3,5-di-tert-Butyl-4-hydroxyphenylpropionic acid | 28 | C_17_H_26_O_3_ | 23.877 | 278.39 |  | 0.237 |
|  | Hexatriacontyl pentafluoropropionate | 29 | C_39_H_73_F_5_O_2_ | 24.010 | 669 |  | 0.248 |
|  | Cyclodecasiloxane, eicosamethyl | 30 | C_20_H_60_O_10_Si_10_ | 24.633 | 741.5394 |  | 0.552 |
|  | **No match** | **31** |  | **24.918** |  |  | **7.0** |
|  | Cyclic octaatomic sulfur | 32 | S_8_ | 25.245 | 256.520 | 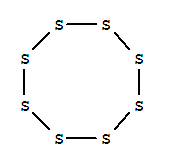 | 0.70 |
|  | Tritetracontane | 33 | C_43_H_88_ | 25.456 | 605.1588 |  | 0.471 |
|  | 1,2-Bis-[2,7-dimethoxyfluoren-9-ylidene]hydrazine | 34 | C_30_H_24_N_2_O_4_ | 26.043 | 476.52256 |  | 3.972 |
|  | 1-Ethyl-3-(pyrrolidin-2-ylidene)-2-indolinone | 35 | C_14_H_16_N_2_O | 26.291 | 228.29 |  | 0.849 |
|  | 3,9.beta.:14,15-Diepoxypregn-16-en-20-one, 3,11.be | 36 | C_21_H_28_O_3_ | 26.409 | 330.21 |  | 0.592 |
|  | dl-Alanine-beta-naphthylamide | 37, 38 | C_13_H_14_N_2_O | 26.665 | 214.26 |  | 5.54 |
|  | Xanthurenic acid, O,O'-bis(tert-butyldimethylsilyl | 39 | C_22_H_35_NO_4_Si_2_ | 26.817 | 433.7 |  | 5.552 |
|  | Pyrazino[1,2-a]indole-1,4-dione, 2,3-dihydro-2-met | 40 | C_13_H_10_ N_2_O_2_ | 27.015 | 226.2307 | 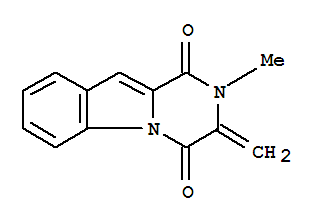 | 13.921 |
|  | 6,6'-Diacetyl-7,7'-dihydroxy-2,2',4,4',5,5'-hexame | 41 | C_24_H_26_O_12_ | 27.562 | 506.45600 | 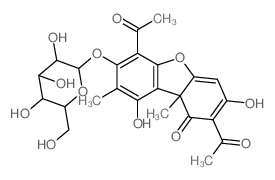 | 2.733 |
|  | 2,3,7,7,12,13,17,18-Octaethyl-21H,23H-porphine-8-o | 42 | C_36_H_46_N_4_O | 27.722 | 550.77700 | 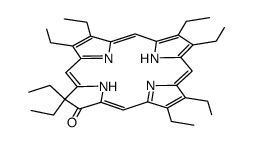 | 4.236 |
|  | l-Leucine, N-(2,3,4-trifluorobenzoyl)-, octyl este | 43 | C_21_H_30_F_3_NO_3_ | 28.207 | 401.46301 |  | 0.421 |
|  | 2,2-Dichloro-1,1-bis(4-methoxyphenyl)ethane | 44 | C_16_H_16_Cl_2_O_2_ | 28.632 | 311.20300 |  | 3.951 |
|  | **No match** | **45** |  | **28.766** |  |  | **3.295** |
|  | Bis(heptamethylcyclotetrasiloxy)hexamethyltrisilox | 46 | C_20_H_60_O_4_Si_11_ | 28.861 | 673.6285 | 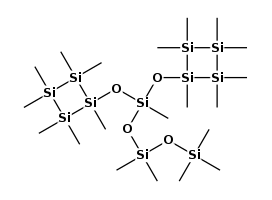 | 3.181 |
|  | 3-Phorbinepropanoic acid, 9-acetyl-14-ethyl-13,14- | 47, 48 | C_29_H_30_N_4_O_3_ | 29.362 | 484.24 |  | 6.05 |
|  | 1,2,3,4-Tetrahydro-6-methylthio-9-acridinol | 49 | C_13_H_14_N_2_ | 30.347 | 245.34 |  | 2.1 |
|  | Bis(pentamethylcyclotrisiloxy)hexamethyltrisiloxan | 50, 51 | [C_16_H_48_O_10_Si_9_](https://pubchem.ncbi.nlm.nih.gov/#query=C16H48O10Si9) | 31.701 | 557.32 |  | 5.82 |

**Table S4: Phytochemical analysis of the compound.**

| **Phytochemical** | **Confirmative Test** | **Observations** | **Inferences** |
| --- | --- | --- | --- |
| Tannins | Bramer’s test | No colour changed in the extract | –– |
| Flavonoids | –– | Formation of white precipitate | –– |
| Terpenoids | –– | No colour changed in the extract | –– |
| Saponins | Foam test | There was no forth | –– |
| Steroid | Salkowski test | Formation of white precipitate | –– |
| Phlobatannins | Precipitate test | No colour changed in the extract | –– |
| Carbohydrates | Molisch’s test | A reddish-violet ring at the junction | + |
| Glycosides | Liebermann’s test | No colour changed in the reaction | –– |
| Coumarins | –– | No yellow colour formed | –– |
| Alkaloids | Hager’s and Wagner's test | Formation of yellow and reddish-brown precipitate respectively | + |
| Proteins | Xanthoproteic test | No colour changed in the reaction | –– |
| Emodins | –– | No colour changed in the reaction | –– |
| Anthocyanins | –– | No colour changed in the reaction | –– |
| Leucoanthocyanins | –– | No colour changed in the reaction | –– |
| Anthraquinones | Borntrager’s test | No colour changed in the reaction | –– |

Note: Here “+” symbol indicates the presence of and “––” absence of the phytochemical group.

**Table S5: Docking scores of the target enzymes/proteins.**

| **PDB Id** | **Enzyme details** | **Functions** | **Docking score** |
| --- | --- | --- | --- |
| **4RUL** | Crystal structure of full-length E. coli topoisomerase I in complex with ssDNA  - **doi:**[10.2210/pdb4RUL/pdb](http://doi.org/10.2210/pdb4RUL/pdb) | Prevents hyper negative supercoiling of DNA of transcription driven negative supercoils  (Tan *et al*. 2015; doi:10.1093/nar/gkv1073) | -6.3 |
| 5YIG | Crystal structure of Streptococcus pneumonia ParE with inhibitor  - **doi:**[10.2210/pdb5YIG/pdb](http://doi.org/10.2210/pdb5YIG/pdb) | Permeability across Gram-negative bacterial membrane as well as bacterial efflux mechanisms.  (Ho *et al.* 2018; doi:10.1016/j.ejmech.2018.08.025) | -5.9 |
| 7DQW | E. coli GyrB ATPase domain in complex with 4-chlorophenol  - **doi:**[10.2210/pdb7DQW/pdb](http://doi.org/10.2210/pdb7DQW/pdb) | ATPase domain of *E. coli* GyrB plays an essential role in regulating DNA topology during transcription and replication.  (Yu *et al.* 2021; doi:10.1016/j.bioorg.2021.105040) | -6.1 |
| 7DQS | E. coli GyrB ATPase domain in complex with 2-chlorophenol  - **doi:**[10.2210/pdb7DQS/pdb](http://doi.org/10.2210/pdb7DQS/pdb) |  | -5.9 |
| 7DQM | E. coli GyrB ATPase domain in complex with Naringenin  - **doi:**[10.2210/pdb7DQM/pdb](http://doi.org/10.2210/pdb7DQM/pdb) |  | -5.7 |
| 7DQL | E. coli GyrB ATPase domain in complex with 4-chlorobenzene-1,2-diol  - **doi:**[10.2210/pdb7DQL/pdb](http://doi.org/10.2210/pdb7DQL/pdb) |  | -5.6 |
| 7DQJ | E. coli GyrB ATPase domain in complex with 3,4-Dihydroxyacetophenone  - **doi:**[10.2210/pdb7DQJ/pdb](http://doi.org/10.2210/pdb7DQJ/pdb) |  | -5.2 |
| 7DQI | E. coli GyrB ATPase domain in complex with Esculetin  - **doi:**[10.2210/pdb7DQI/pdb](http://doi.org/10.2210/pdb7DQI/pdb) |  | -5.9 |
| 7DQF | E. coli GyrB ATPase domain in complex with methyl 2,4-dihydroxybenzoate  - **doi:**[10.2210/pdb7DQF/pdb](http://doi.org/10.2210/pdb7DQF/pdb) |  | -5.4 |
| 7DPS | E. coli GyrB ATPase domain in complex with Methyl 4-hydroxycinnamate  - **doi:**[10.2210/pdb7DPS/pdb](http://doi.org/10.2210/pdb7DPS/pdb) |  | -6.0 |
| 7DPR | E. coli GyrB ATPase domain in complex with methyl 3,4-dihydroxybenzoate  - **doi:**[10.2210/pdb7DPR/pdb](http://doi.org/10.2210/pdb7DPR/pdb) |  | -5.9 |
| 7DOR | E. coli GyrB ATPase domain in complex with 4-nitropheno  - **doi:**[10.2210/pdb7DOR/pdb](http://doi.org/10.2210/pdb7DOR/pdb) |  | -4.8 |
| 7DQH | E. coli GyrB ATPase domain in complex with 2-hydroxybenzamide  - **doi:**[10.2210/pdb7DQH/pdb](http://doi.org/10.2210/pdb7DQH/pdb) |  | -6.3 |
| 1KSE | Solution Structure of a quinolone-capped DNA duplex  - **doi:**[10.2210/pdb1KSE/pdb](http://doi.org/10.2210/pdb1KSE/pdb) | Quinolones are gyrase inhibitors. Quinolones block re-ligation of DNA strands in the active site of gyrases.  (Tuma *et al.* 2002; doi:10.1021/ja0125117) | -5.5 |
| 6JX9 | Structure of Y17107 complexed HPPD  - **doi:**[10.2210/pdb6JX9/pdb](http://doi.org/10.2210/pdb6JX9/pdb) | 4-hydroxyphenylpyruvate dioxygenase (HPPD, EC 1.13.11.27) is a potential target for herbicides  (He *et al.* 2019; doi:10.1021/acs.jafc.9b04917) | -6.2 |
| 3W9H | Structural basis for the inhibition of bacterial multidrug exporters  - **doi:**[10.2210/pdb3W9H/pdb](http://doi.org/10.2210/pdb3W9H/pdb) | Principal multidrug exporters in *Pseudomonas aeruginosa*8–10.  (Nakashima *et al.* 2013; doi:10.1038/nature12300) | -6.1 |
| 2LQV | YebF  - **doi:**[10.2210/pdb2LQV/pdb](http://doi.org/10.2210/pdb2LQV/pdb) | *E. coli* protein transport  (Prehna *et al.* 2012; doi:10.1016/j.str.2012.04.014) | -5.8 |
| 5NUQ | Structural basis for maintenance of bacterial outer membrane lipid asymmetry  - **doi:**[10.2210/pdb5NUQ/pdb](http://doi.org/10.2210/pdb5NUQ/pdb) | The Gram-negative bacterial outer membrane (OM) lipoprotein MlaA that selectively removes outer leaflet phospholipids to help maintain the essential barrier function of the bacterial OM.  (Abellón-Ruiz *et al*. 2017; doi: 10.1038/s41564-017-0046-x) | -5.8 |
| 5NUR | Structural basis for maintenance of bacterial outer membrane lipid asymmetry  - **doi:**[10.2210/pdb5NUR/pdb](http://doi.org/10.2210/pdb5NUR/pdb) |  | -5.3 |
| 5NUP | Structural basis for maintenance of bacterial outer membrane lipid asymmetry****doi:****[10.2210/pdb5NUP/pdb](http://doi.org/10.2210/pdb5NUP/pdb) |  | -6.2 |
| 5O79 | Klebsiella pneumoniae OmpK36  - **doi:**[10.2210/pdb5O79/pdb](http://doi.org/10.2210/pdb5O79/pdb) | The principal general porins of Enterobacteriaceae that determine antibiotic permeabilities  (Acosta-Gutiérrez *et al.* 2018; doi:10.1021/acsinfecdis.8b00108) | -6.2 |
| 5O77 | Klebsiella pneumoniae OmpK35  - **doi:**[10.2210/pdb5O77/pdb](http://doi.org/10.2210/pdb5O77/pdb) |  | -5.5 |
| 4JFB | Crystal structure of OmpF in C2 with tNCS  - **doi:**[10.2210/pdb4JFB/pdb](http://doi.org/10.2210/pdb4JFB/pdb) | The outer membrane protein OmpF of *E. coli*  (Wiseman *et al.* 2014; doi:10.1371/journal.pone.0114864) | -1.6 |
| 3NB3 | The outer membrane proteins OmpA and OmpC of Shigella phage Sf6 virion.****doi:****[10.2210/pdb3NB3/pdb](http://doi.org/10.2210/pdb3NB3/pdb) | Outer membrane protein (OMP) porins for the lysogenic cycle.  (Zhao *et al.* 2011; doi:10.1016/j.virol.2010.10.030) | -4.8 |
| 1KZN | E. coli 24 kDa domain in Complex of DNA gyrase.  - **doi:**[10.2210/pdb1KZN/pdb](http://doi.org/10.2210/pdb1KZN/pdb) | [DNA Gyrase subunit B](https://www.rcsb.org/search?request=%7B%22query%22%3A%7B%22type%22%3A%22terminal%22%2C%22service%22%3A%22text%22%2C%22parameters%22%3A%7B%22attribute%22%3A%22rcsb_polymer_entity.pdbx_description%22%2C%22operator%22%3A%22contains_phrase%22%2C%22value%22%3A%22DNA%20GYRASE%20SUBUNIT%20B%22%7D%7D%2C%22return_type%22%3A%22entry%22%7D)(protein)  (Lafitte *et al.* 2002; doi:10.1021/bi0159837) | -4.8 |
| 6YD9 | E. coli GyrB24 with inhibitor 16a  - **doi:**[10.2210/pdb6YD9/pdb](http://doi.org/10.2210/pdb6YD9/pdb) | DNA gyrase and DNA topoisomerase IV of Gram-negative bacteria.  (Skok *et al*. 2020; doi:10.1021/acsmedchemlett.0c00416) | -6.2 |
| 4KPE | Topoisomerase IV from S. pneumoniae  - **doi:**[10.2210/pdb4KPE/pdb](http://doi.org/10.2210/pdb4KPE/pdb) | Topoisomerase IV relaxes supercoiled DNA; is essential for chromosome segregation.  (Leo et al. 2005; doi:10. 1074/jbc.M500156200; Laponogov *et al*. 2016; doi:10.1098/rsob.160157) | -5.9 |
| 3RAD | Type IV topoisomerase from S. pneumoniae  - **doi:**[10.2210/pdb3RAD/pdb](http://doi.org/10.2210/pdb3RAD/pdb) |  | -6.0 |
| 1KF6 | E. coli Quinol-Fumarate Reductase **doi:**[10.2210/pdb1KF6/pdb](http://doi.org/10.2210/pdb1KF6/pdb) | The quinol-fumarate reductase (QFR) is a respiratory complex that catalyzes the final step of anaerobic respiration  (Iverson *et al.* 2002; doi:10.1074/jbc.m200815200) | -7.0 |
| 1L0V | Quinol-Fumarate Reductase with Menaquinol Molecules  - **doi:**[10.2210/pdb1L0V/pdb](http://doi.org/10.2210/pdb1L0V/pdb) |  | -7.1 |
| 2WP9 | E. coli succinate:quinone oxidoreductase (SQR) ****doi:****[10.2210/pdb2WP9/pdb](http://doi.org/10.2210/pdb2WP9/pdb) | Succinate-ubiquinone oxidoreductase (SQR) is a member of the complex II family of enzymes.  (Ruprecht *et al*. 2011; doi:10.1074/jbc.m110.209874) | -6.9 |
